# The Dermal Skeleton of Stem‐Actinopterygian *Moythomasia durgaringa* and Its Implications for the Nature of the Ancestral Osteichthyan

**DOI:** 10.1002/jmor.70120

**Published:** 2026-03-19

**Authors:** Xianren Shan, Edine Pape, Joseph N. Keating, Martin Rücklin, Davide Pisani, Philip C. J. Donoghue

**Affiliations:** ^1^ Bristol Palaeobiology Group, Schools of Earth and Biological Sciences University of Bristol Bristol UK; ^2^ Hezelburcht Grant and Funding Consultancy Nijmegen the Netherlands; ^3^ Naturalis Biodiversity Center Leiden the Netherlands; ^4^ Institute of Biology Leiden Leiden University Leiden the Netherlands

## Abstract

The dermal skeleton is the most primitive component of the vertebrate mineralized skeleton, and features of its structure and development are key to resolving the evolutionary relationships of early vertebrates and bony fishes. In particular, the nature and phylogenetic distribution of cosmine, a dermal complex of hard tissue and vascular systems, have been the focus of debate over the nature of the ancestral osteichthyan and the timing of actinopterygian–sarcopterygian divergence. In large part, this controversy occurs because of a paucity of knowledge of the nature of the dermal skeleton in stem‐actinopterygians. Here, we describe the dermal skeletal histology of stem‐actinopterygian *Moythomasia durgaringa* using Scanning Electron Microscopy and Synchrotron Radiation X‐ray Tomographic Microscopy with a reconstruction of its topological variation and development. The dermal skeleton of *Moythomasia* consists of a superficial layer of stacked odontodes that undergo extensive odontogenic resorption and a basal layer of lamellar bone. A middle vascular bone layer is variably developed in cranial dermal bones but is completely absent in postcranial dermal elements. Additional histological variation among dermal elements includes the number of odontode generations, odontode growth patterns and the relative thickness of osteogenic and odontogenic tissues. A comparison of the histological condition in *Moythomasia* and stem‐ and early crown‐osteichthyans reveals numerous similarities, including the presence of a three‐layered dermal skeleton, stacked odontodes and odontogenic resorption. Phylogenetic comparative analyses on early jawed vertebrates indicate that features associated with cosmine evolved in groups outside Rhipidistia, whereas true cosmine remains restricted to this group comprising Dipnomorpha and Tetrapodomorpha. The concept of cosmine is phylogenetically uninformative because of the multiplicity of its definitions and usage. These findings suggest that fossil taxa currently classified as stem‐sarcopterygians may instead be stem‐actinopterygians, or even stem‐osteichthyans, with implications for the nature of the ancestral bony fish and the timing of osteichthyan diversification.

## Graphical abstract

The figure presents a model of *Moythomasia* and a schematic histological model illustrating the internal structure and features of the cranial bones. These include bone (brown), osteocyte spaces (red), spheritic bone (light brown), osteon spaces (orange), dentine and canaliculi (green), pulp canal (yellow), ganoine (gray), arrested growth lines (dashed lines) and resorption lines (white). *Moythomasia* reconstruction credited to Brian Choo.

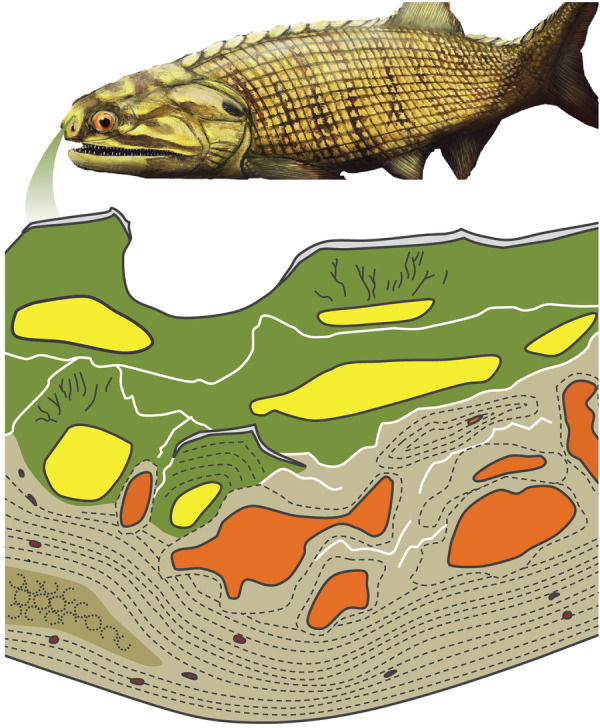

## 1 Introduction

**Figure 1:**
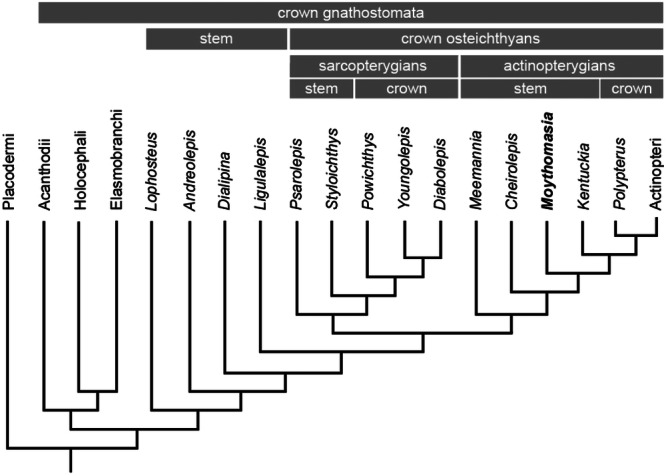
Phylogenetic relationships of jawed vertebrates and the phylogenetic position of *Moythomasia*. Simplified from Lu et al. (2016) and Giles et al. (2017).

The vertebrate dermal skeleton is one of the key innovations that has underpinned the explosive diversification of the vertebrate lineage. This skeletal system first evolved in ancient stem‐gnathostomes as a stratification of mineralized tissues and underwent major morphological and histological modifications in modern lineages, particularly bony fishes (Sire et al. 2009). Reconstruction of the evolution of the dermal skeleton and its canonical tissue types (dentine, enamel and bone) is essential for resolving the evolution of the vertebrate skeleton and the evolutionary relationships of early vertebrates. Osteichthyes are of particular significance, as bone has largely been lost from chondrichthyans and because this clade of bony fishes, comprised of Actinopterygii (ray‐finned fishes) and Sarcopterygii (lobe‐finned fishes), encompasses the vast majority of vertebrate diversity. However, the nature of the dermal skeleton in early osteichthyans remains poorly understood, because of debate on the nature and phylogenetic distribution of cosmine, a dermal complex of hard tissue and vascular systems (Goodrich 1907, 1909; Ørvig 1969). This controversy occurs principally because of a paucity of knowledge of the nature of the dermal skeleton in stem‐actinopterygians. Recent cladistic studies have reassigned numerous Paleozoic ray‐finned fishes from the actinopterygian crown to the stem (Giles et al. 2017), providing a new framework to infer the ancestral actinopterygian condition (Figure 1). Despite their importance for understanding the dermal skeletal evolution in osteichthyans, the detailed histology of stem‐actinopterygians remains poorly investigated. Previous histological studies on stem‐actinopterygians have mainly focused on individual scales (Ørvig 1978; Gardiner 1984; Zylberberg et al. 2015), with few investigations of cranial bones (Lu et al. 2016), even though variation is known to exist across the dermal skeleton. To establish a model for interpreting actinopterygian dermal histology in fossil species, we undertook a detailed histology study of the dermal skeleton in *Moythomasia durgaringa*, encompassing topological variation across the head and trunk. We focussed on *M. durgaringa* because it is one of the earliest known members of the actinopterygian stem group and because it is known from numerous exceptionally preserved specimens from the Late Devonian Gogo fossil Lagerstätte (Trinajstic et al. 2022). *M. durgaringa* has long been interpreted as an early crown‐actinopterygian (e.g., Janvier 1996) but with the reclassification of the extant polypterids among extinct scanilepiforms, *M. durgaringa* has been resolved as an early stem‐actinopterygian (Giles et al. 2017). As such, with comparison to other stem‐ and crown‐actinopterygians, analysis of *M. durgaringa* has the potential to provide information on the nature of the dermal skeleton in early actinopterygians more generally. We characterized the histology of diverse dermal skeletal elements from a number of articulated specimens of *M. durgaringa* using scanning electron microscopy (SEM) and synchrotron radiation X‐ray tomographic microscopy (SRXTM).

### 1.1 Previous Histological Studies of Stem‐Actinopterygians

Among stem‐actinopterygians, there are only three genera *Cheirolepis*, *Moythomasia* and *Mimipiscis*, that have been studied histologically (Gardiner 1984; Zylberberg et al. 2015; Lu et al. 2016). Several late Silurian–Early Devonian taxa (*Dialipina*, *Ligulalepis*, *Meemannia*, and *Psarolepis*) have been variously resolved as stem‐osteichthyans, stem‐actinopterygians or stem‐sarcopterygians, with little consensus regarding their relationships (Zhu et al. 2009; Dupret et al. 2014; Long et al. 2015; King et al. 2017; Lu et al. 2017). Of these taxa, histological studies have focused on the dermal skull of *Meemannia* (Zhu et al. 2006, 2010; Lu et al. 2016), the dermal scales and skull of *Psarolepis* (Qu et al. 2013b, 2017), and the dermal scales of *Dialipina* and *Ligulalepis* (Schultze 1968). Dermal scales of stem‐actinopterygians (*Cheirolepis*, *Moythomasia*, and *Mimipiscis*) are histologically similar to each other (Zylberberg et al. 2015), consisting of a superficial layer of ganoine overlying dentine and a thick bone attachment, classified as “palaeoniscoid scales” (an early type of ganoid scale, Goodrich 1907; Sire et al. 2009). Meanwhile, the histology of cranial dermal bones of stem‐actinopterygians has been understudied. Studies on the 2D section of the skull roof of *Meemannia* and *Cheirolepis* revealed that they consist of lamellar bone overlain by a cosmine‐like layer consisting of superimposed layers of odontodes and a pore canal network opening to the surface (Lu et al. 2016). Despite these observations, the pattern of histological variation across the dermal skeleton in stem‐actinopterygians remains poorly understood.

For the two osteichthyan clades, a main distinction is the presence of ganoine in the actinopterygian dermal skeleton and cosmine within sarcopterygians. Cosmine is a hard tissue complex (enamel, dentine, and bone of attachment) that includes a set of canals known as the pore canal system (Gross 1956; Ørvig 1969). For a detailed review of cosmine, refer to Schultze (2016) and Mondéjar‐Fernández (2018). *Meemania* was originally described as a stem‐sarcopterygian based on the presence of a pore canal system (Zhu et al. 2006). However, Lu et al. (2016) re‐examined *Meemania* using X‐ray tomography and identified several key actinopterygian features, including the endoskeletal enclosure of the spiracle and a lateral cranial canal. They also reported evidence of a pore canal system in *Cheirolepis*, an unequivocal actinopterygian taxon. Based on these observations, Lu et al. (2016) revised a previous phylogenetic matrix and updated the character coding for *Meemania* and *Cheirolepis*, analysis of which placed *Meemania* among stem‐actinopterygians. This result implies that the pore canal system, once regarded as a defining feature of sarcopterygians, is more widely distributed across early bony fishes. However, there are many different definitions of cosmine, in terms of the structure and function of the pore canal system, layers of enamel and odontogenic resorption, with concepts including “eucosmine” and “proto‐cosmine” (Thomson 1975, 1977; Schultze 2016; Lu et al. 2016; Qu et al. 2017; Mondéjar‐Fernández 2018; Cui et al. 2025). With this multiplicity of concepts and definitions, cosmine has been variously recognized in early osteichthyans such as *Meemannia*, *Cheirolepis*, and *Psarolepis*, which consequently impact the classification of these taxa, whether as stem‐osteichthyans, stem‐sarcopterygians or stem‐actinopterygians (Schultze 2016; Lu et al. 2016; Mondéjar‐Fernández 2018). Within this debate, the concept of cosmine has been blurred to become phylogenetically meaningless. Here, we test the validity of synapomorphic status of cosmine through analysis of the dermal skeleton of the stem‐actinopterygian *Moythomasia durgaringa* and evaluate the phylogenetic distribution of cosmine‐related characters within the osteichthyans.

## 2 Materials and Methods

### 2.1 Materials

**Figure 2:**
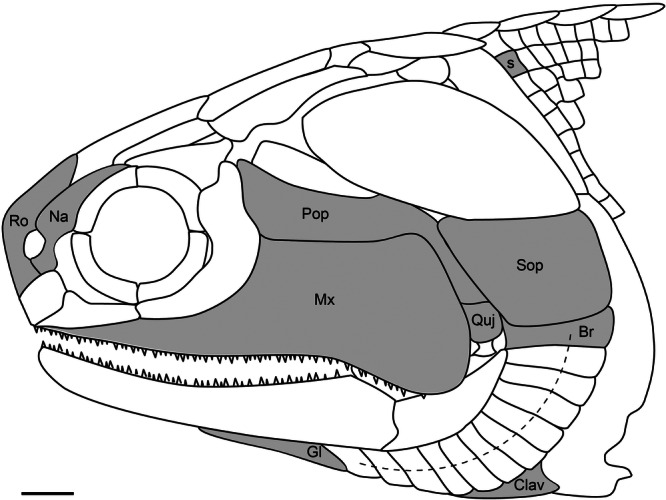
Restoration of the cranial and part of the postcranial dermal skeleton of *Moythomasia durgaringa* (Gardiner 1984), showing the dermal elements (in gray) used in this study. Br, Branchiostegal; Clav, Clavicle; Gl, Gular; Mx, Maxillary; Na, Nasal; Pop, Preopercular; Quj, Quadratojugal; Ro, Rostral; S, Scale; and Sop, Subopercular. Scale bar = 2 mm.

This study is based on the Late‐Devonian *Moythomasia durgaringa* from the Late Devonian Gogo Formation of Western Australia, reposited at the Natural History Museum, London (NHMUK). Elements from a wide range of anatomical positions and developmental stages were investigated (Figure 2). Early developmental stages were identified by their very small size, translucency due to incomplete ossification and lack of adult features. The intermediate developmental stage was determined on the basis of size. The four specimens analyzed include NHMUK PV P.53221 (late stage), NHMUK PV P.56502 (late stage), NHMUK PV P.53219 (intermediate stage) and NHMUK PV P.56425 (early stage). Detailed histology of the elements was assessed using scanning electron microscopy (SEM) and synchrotron radiation X‐ray tomographic microscopy (SRXTM). The elements analyzed using SEM include trunk scales (P.53221, P.56425), branchiostegal rays (P.53221), gular plates (P.53219), hypobranchial plates (P.56502), subopercular plates (P.56502), preopercular/quadratojugal plates (P.53219), maxillary plates (P.56425) and the clavicle (P.56425). The elements analyzed using SRXTM include trunk scales (P.53221, P.56425), branchiostegal rays (P.53221, P.56425), nasal plates (P.53221, P.56425) and rostral plates (P.53221, P.53219).

### 2.2 Methods

#### 2.2.1 Synchrotron Radiation X‐ray Tomographic Microscopy

Volumetric characterization of the histology of the dermal elements was carried out by three‐dimensional reconstruction of elements using SRXTM (Donoghue et al. 2006a) at the X02DA TOMCAT beamline of the Swiss Light Source, Paul Scherrer Institut, Villigen, Switzerland (Bonnin et al. 2024). Specimens were mounted on 3.1 mm diameter pins using nail varnish and analyzed using beam current of 10–28 KeV, 120–1200 ms exposure, a 20 µm LuAG:Ce scintillator and 2×, 4×, 10× and 20× objectives, yielding reconstructed tomographic data with voxel dimensions of 3.25, 1.625, 0.65, and 0.325 µm, respectively. For each scan, 1501 projections were taken equi‐angularly through 180° rotation within the beam. Projections were post‐processed and rearranged into flat‐ and dark‐field–corrected sinograms, and reconstruction was performed on a 60‐core Linux PC farm, using a highly optimized routine based on the Fourier transform method and a regridding procedure (Marone et al. 2017). Analysis, segmentation and 3D visualization of the tomographic data were performed in Dragonfly software, version 2024 (www.theobjects.com/dragonfly). The histology structure of the dermal skeletal tissues was resolved on the basis of differential X‐ray attenuation.

#### 2.2.2 Scanning Electron Microscopy

SEM analysis irradiates the sampled surface with electrons, whereby the strength of backscattered electrons (BSEs) reflects elemental make‐up. SEM analysis was carried out on a Hitachi S‐3500N at the Electron Microbeam Facility of the Department of Earth Sciences, University of Bristol. Because of the destructive nature of the method, specimens were photographed with a stereomicroscope (Leica M205C) using a digital microscope camera (DFC425C). For SEM analysis, skeletal elements were embedded in the cold‐curing transparent polyester resin Buehler Epothin and sectioned using a Buehler Isomet low‐speed saw. The cut faces were impregnated with Buehler Epothin, ground on silicon carbide paper (800 and 1200 µm) and polished on paper laps using Buehler MetaD Diamond Paste (6 and 1 µm) mixed with Buehler IsoCut Fluid for lubrication. Elements were coated in gold before SEM analysis.

## 3 Results

The dermal skeleton of *Moythomasia durgaringa* consists of three tissue layers, with contributions from these tissue layers varying across the dermal elements. The superficial layer consists of overlapping odontodes forming the external ornament. Each odontode is composed of a bone of attachment supporting a core of dentine that is pervaded by radially polarized canaliculi and overlain by a hypermineralized capping tissue. The middle layer consists of cellular vascular bone containing numerous canals, variously developed in cranial dermal bones. The basal layer is composed of lamellar bone with sparsely distributed lacunae. A complete description of the condition of the scales is presented; remarks on dermal elements from the other skeletal regions are limited to deviations from this general condition. An ontogenic stage is indicated only if the element does not represent the late developmental condition.

### 3.1 Histological Structure of the Postcranial Dermal Skeleton

**Figure 3:**
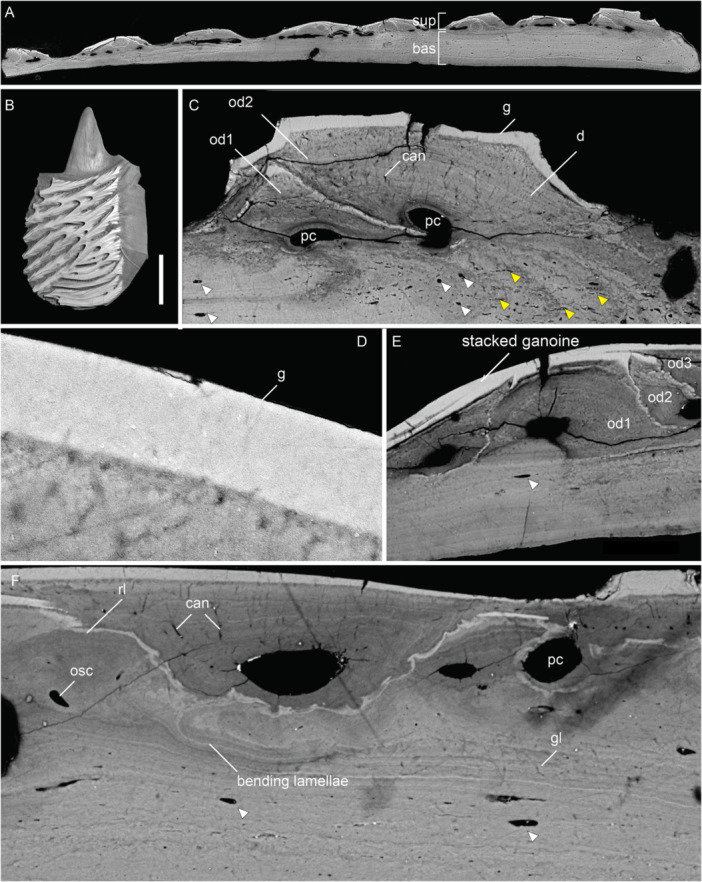
Scales of *Moythomasia durgaringa*. (A) A transverse section showing the composition of (B) an adult scale (NHMUK PV P.53221). (C) Composition of an odontocomplex and underlying bone at the edge of a scale; arrowheads in white indicate osteocyte lacuna and arrowheads in yellow indicate spaces of Sharpey's fibers. (D) Detail of ganoine with underlying dentine, in the transverse section. (E) Overview of the divisions in the longitudinal section showing the stacked ganoine and a clear boundary between the superficial and basal layers. (F) Resorption within the superficial layer in the transverse section. bas, basal layer; can, canaliculi; d, dentine; g, ganoine; lb, lamellar bone; od, odontode; osc, osteocyte; pc, pulp canal; rl, resorption line; and sup, superficial layer. Scale bar = 216 µm (A), 1000 µm (B), 34 µm (C), 6 µm (D), 32 µm (E) and 18 µm (F).

The postcranial dermal elements examined include scales (250–375 µm thick) and a clavicle (81–202 µm thick) from the pectoral girdle. Scales studied belong to the dorsal anterior area of body covering, ornamented with diagonal ridges. The gross composition of scale consists of a basal division of lamellar tissue and a superficial division of stacked odontodes forming the external ornamental ridges (Figure 3A,B).

**Figure 4:**
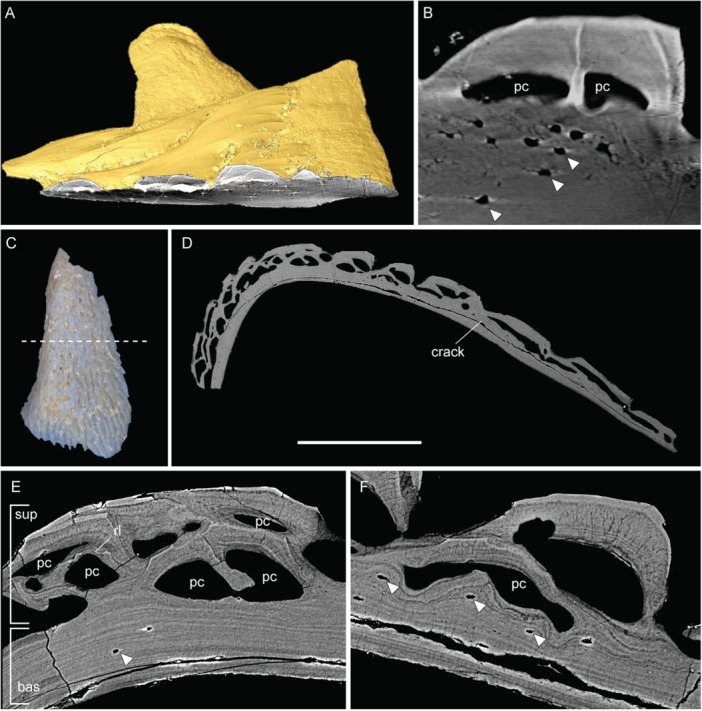
Juvenile scale and clavicle of *Moythomasia durgaringa*. (A) Surface rendering (gold) of a juvenile scale (NHMUK PV P.56425) with a transverse section showing the SRXTM image. (B) SRXTM image of (A) showing large pulp canals and osteocyte lacunae (arrowheads). (C) Photo of a juvenile clavicle (NHMUK PV P.56425) in the dorsal view. (D–F) SEM images, a crack is present throughout the element. (D) A transverse section showing the composition of the dermal skeleton. (E) Overview of the divisions, lacking a middle layer. (F) Superficial layer influencing the basal layer and the presence of osteocytes (an artifact is present next to the pulp canal). bas, basal layer; pc, pulp canal; and sup, superficial layer. Scale bar = 625 µm (A), 108 µm (B), 2295 µm (C), 656 µm (D), 103 µm (E) and 100 µm (F).

The superficial layer (15% total thickness in the center of the scale) consists of vertically stacked odontodes, and each individual odontode consists of a pulp canal surrounded by a core of dentine capped with a hypermineralized tissue (Figure 3C), all supported by a bone of attachment fused to the underlying basal bony plate. Dentine is layered in a concentric manner, reflecting centripetal growth layers infilling the pulp canal, and contains canaliculi orientated perpendicular to the dentine growth layers (Figure 3C). The tissue is identified more specifically as orthodentine based on the presence of approximately parallel polarized canaliculi and the absence of cell lacunae within the mineralized tissue. Pulp canals follow the contour of the odontode, with a diameter of 25–50 μm in adult scales, whereas juvenile scales show larger pulp canals (Figure 4A,B), reaching 100–150 μm in diameter. The hypermineralized tissue is typically a single layer with a thickness of 5–10 μm, tapering towards the margins (Figure 3C,D). In regions where odontodes are closely packed, multiple layers of hypermineralized tissue occur without intervening dentine (Figure 3E). This tissue has been identified as ganoine (Gardiner 1984), which is generally considered homologous with the enamel (Sire et al. 1987; Zylberberg et al. 1997; Qu et al. 2015; Kawasaki et al. 2021).

In buried odontodes, odontogenic resorption is extensive, displaying loss of the ganoine, to a lesser extent of the underlying dentine. Th*e* process of resorption has left a characteristic morphology of thin, scalloped resorption lines that follow the shape of the overlying odontode (Figure 3F). Part of the pre‐existing odontode is resorbed, leaving a resorption bay occupied by part of the overlying new odontode, a process successively repeated when an odontode is added. The superficial layer is composed of different generations of odontodes deposited throughout the ontogeny, collectively known as an odontocomplex (Ørvig 1969).

The basal layer comprises almost the complete thickness in the middle region of the scale (approximately 85% total thickness), albeit becoming thinner towards the margin of the scale. It consists of parallel lamellae with sparsely distributed lacunae (Figure 3C,E,F) and a few vascular canals. Lacunae lie within and between the lamellae and are often round, with a slight tendency towards an elongate shape, with long axes parallel to the lamellae, in the most superficial lamellae (Figure 3C,F). In the basal most lamellae, however, lacunae are extremely rare (Figure 3A). The lamellae show different brightnesses in the X‐ray tomographic data, reflecting differential X‐ray attenuation, and represent lines of arrested growth (Figure 3F). Individual lamellae are average 10 µm in thickness. Sharpey's fibers for attachment occur within the basal layer, extending through the lower surface of the scale (Figure 3C). In general, the distinction between the superficial and basal layers is sharp (Figure 3E), whereas curved lamellae are observed directly underlying resorption areas (Figure 3F). Resorption within the superficial layer also impacts on the underlying attachment bone.

Scales in the early developmental stage (Figure 4A) show a very similar composition, except for the thin basal division (measuring 50–60 μm) and large pulp canals (100–150 μm in diameter). In the basal layer of juvenile scales, lacunae are large and rounded, with small ramifying branches that may represent osteocyte processes (Figure 4B).

The histology of juvenile clavicle (Figure 4C) shows a similar composition to the scale, consisting of a superficial layer of odontodes of numerous generations and a basal layer of lamellar bone (Figure 4D–F). The boundary between the superficial and basal layers is generally smooth (Figure 4E). Osteocytes are scarce within the basal layer of the clavicle, although they tend to be more abundant in areas showing active growth (Figure 4F).

**Figure 5:**
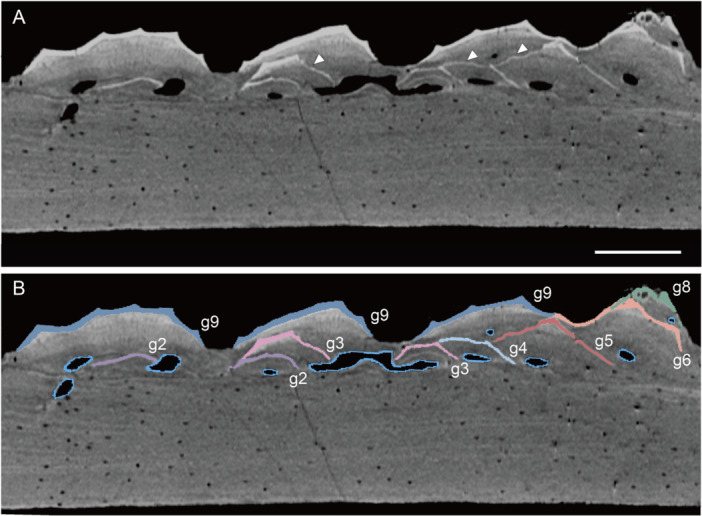
Segmentation of virtual thin sections of *Moythomasia* scales. (A) Virtual section cutting vertically through the ornamental ridges of an adult scale (NHMUK PV P.53221) showing stacked odontodes and vascular canals; note resorption to the enamel layers (arrowhead). (B) The same virtual thin section as in (A). Different generations of odontodes (g2–g10) are represented by different enamel layers marked by different regions of interest (ROIs). Scale bar = 50 µm.

**Figure 6:**
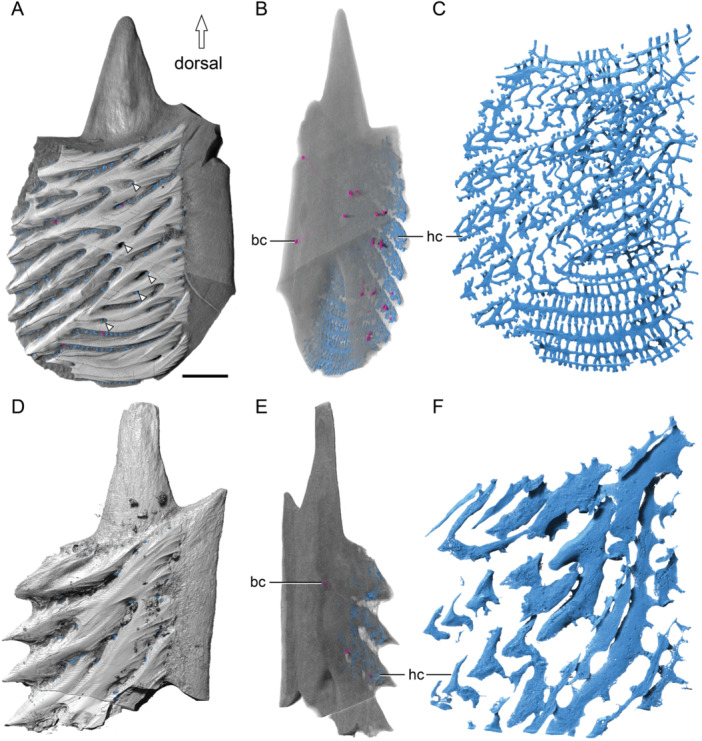
Vascular canal of scales of *Moythomasia durgaringa*. (A, D) Dorsal views of an adult scale (A) and a juvenile scale (D); arrowheads indicate surface opening penetrating ganoine. (B, E) Basal views of an adult scale (B) and a juvenile scale (E), showing the position of basal canals; hard tissue is shown as transparent. (C, F) Reconstruction of the vascular canals in an adult scale (C) and a juvenile scale (F). bc, basal canal; hc, horizontal canal. Scale bar = 1000 µm (A, B), 720 µm (C), 650 µm (D, E) and 300 µm (F).

Three‐dimensional reconstruction reveals that all the canals within the scale are interconnected, forming an integrated system (Figures 5, 6C). The system opens to the surface of the scale through numerous pores: 17 pores at the base (Figure 6B), five pores on the surface ridges through the enamel layer (arrowheads, Figure 6A) and many others between the ridges through the bony tissues. The five pores that open on the crown surface piercing the dental tissues (arrowheads, Figure 6A) are much larger in diameter than the other pores. The canal system can be subdivided into two parts: a basal part, comprising vertical basal canals (bc, Figure 6B) within the bony base, and a superficial part, consisting of horizontal canals (hc, Figure 6C) underlying the odontodes and giving rise to polarized canaliculi. The bony base contains 17 vertical canals connecting with the superficial horizontal canals. The superficial horizontal canal system extends along the ridges. The topology of each horizontal canal follows the shape of the overlying odontodes. For long ridge‐like odontodes, the underlying horizontal canals are elongated and connected by numerous perpendicularly oriented narrow side canals (sc, Figure 6C, F) that show semi‐regular spacing. Horizontal canals supplying different generations of odontodes are interconnected. Within short, triangular odontodes, side canals (sc) branch from horizontal canals and extend along the direction of odontode addition (Figure 6C). It is noteworthy that horizontal canals within the same generation are not generally directly connected. In some spaces, narrow connecting canals open onto the external surface of the scale through a ring of foramina around the base of each odontode (Figure 6A). The vascular canals in juvenile scale show a similar organization, except that they have fewer basal canals and larger horizontal pulp canals (Figure 6D–F).

### 3.2 Growth of the Scale

The stacking of individual odontodes is interpreted as successive generations deposited throughout ontogeny. Accordingly, the growth history of the superficial layer can be reconstructed by tracing the sequence of buried odontodes, provided that odontogenic tissues have not been completely resorbed. In the studied material, each odontode surface was reconstructed by segmenting its continuous enamel layer and associated resorption line in serial virtual sections of SRXTM data. Successive generations of odontodes are distinguished by their overlapping relationships, with older generations completely or partially covered by the younger ones (Figure 5). In cases where multiple odontodes collectively overlie the same generation without overlapping one another, they are identified as belonging to the same generation (Figure 5). Therefore, each generation consists of multiple odontodes, and the sequence of odontode addition within a generation cannot be further determined. The lamellae of the bony base are distinguished by differential X‐ray attenuation, representing lines of arrested growth. Four significant growth lines are traced and segmented in 3D histological data, which are hypothesized to represent the major stages of bone growth. These criteria allowed us to reconstruct the complete sequence of ontogenetic events and to observe the relationship between bone growth and odontode addition. Our data indicate that growth of the superficial odontogenic tissue and basal bone attachment is distinct but coordinated, such that each generation of odontodes is added in coordination with the addition of basal bony tissue.

**Figure 7:**
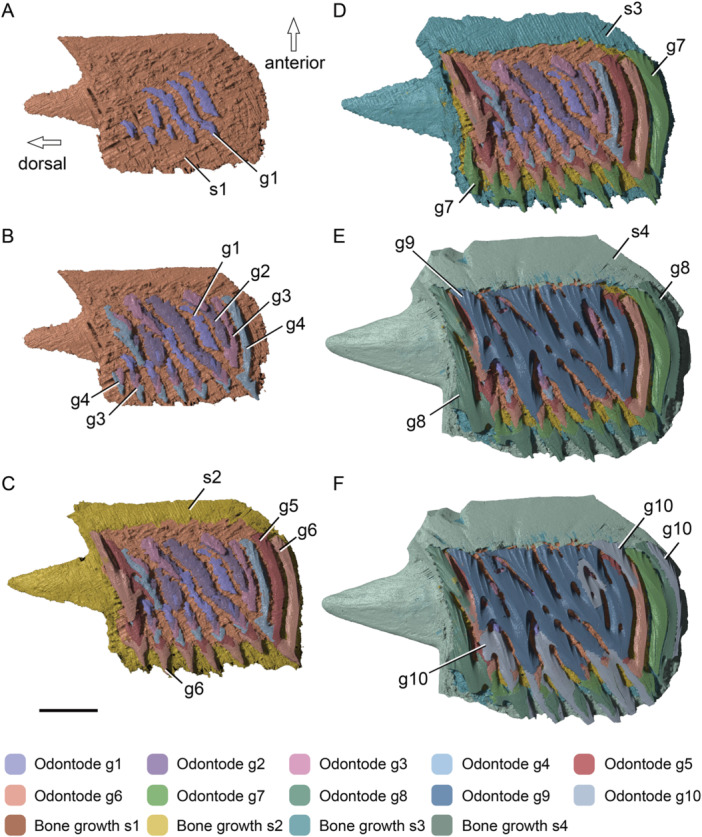
Reconstruction on the growth of an adult scale of *Moythomasia durgaringa*. (A, B) Bone growth stage 1 (s1) with the overlying odontode generation 1 (g1, A) and generations 2–4 (g2–g4, B). (C) Bone growth stage 2 (s2) with the overlying odontode generations 5 and 6 (g5, g6). (D) Bone growth stage 3 (s3) with the overlying odontode generation 7 (g7). (E) Bone growth stage 4 (s4) with the overlying odontode generations 8 and 9 (g8, g9). (F) Bone growth stage 4 (s4) with the overlying odontode generation 10 (g10); note that g9 is a large odontode cover with several surface openings penetrating the ganoine. Scale bar = 1000 µm.

The superficial layer of the studied scale is composed of 10 generations of odontodes (Figure 7), of which the last five generations are partially or completely exposed to the scale surface, whereas the first five generations are fully embedded inside the scale crown. Each generation consists of either multiple single odontodes or a large odontode cover. Four significant growth stages of bone growth are distinguished. Odontodes of generations 1–4 (g1–4) are attached to the basal bone attachment of the first growth stage (s1, Figure 7A,B). Odontodes of generation 1 (g1) grow at the center of the bone base and consist of seven separate odontodes. Each odontode is generally ridge‐like with variable length, even though its original morphology is largely modified by the odontogenic resorption. The resorption process resulted in the enamel layer fading away laterally in 3D histological data, resulting in a serrated margin on the reconstructed enamel surface. Each odontode becomes narrower towards the apex and ends with a sharp pointed tip. Odontodes in g2 are composed of seven separate odontodes. Among them, five odontodes are triangular, and they partially overlap the tips of odontodes of g1 and extend along the ridges, showing a polarized growth pattern. One odontode of g2 completely covers that of g1, with extensions in both directions along the ridges. Another odontode forms a new ridge along the lateralmost odontode of g1. Odontodes in g3–g4 are added in a similar pattern to odontode g2, with several triangular odontodes partially overlapping the tips of the last generation and a new ridge‐like odontode forming in a position lateral to previous odontodes.

Odontodes of g5 and g6 are attached to the attachment bone of the second growth stage (s2, Figure 7C), odontodes of g7 are attached to that of the third growth stage (s3, Figure 7D) and the last three generations (g8–10) are attached to that of the fourth stage (s4, Figure 7E,F). Odontodes of g5–8 are added in a similar pattern to previous generations. Odontode g9 is composed of an extensive and continuous single sheet of enamel overlying dentine and is therefore interpreted as a single odontode (Reif 1982). This large odontode covers most of the pre‐existing odontodes and is pierced by four pores (Figure 7E). Odontodes of g10 contribute both to the expansion of the scale surface and to the infilling of gaps between ornamental ridges (Figure 7F). They are shaped like random patches added to the margins of pre‐existing odontodes that remain uncovered by odontodes of g9.

### 3.3 Histological Structure of Cranial Dermal Skeleton

Several elements of the cranial dermal skeleton of *Moythomasia durgaringa* were analyzed, including the nasal, rostral, preopercular, quadratojugal, maxillary, branchiostegal, gular and subopercular plates. These dermal elements show distinct patterns of external ornamentation: a smooth surface with round pits in the branchiostegal rays and gular plates; short, aligned ridges in the subopercular plates; dashed ridges in the preopercular and quadratojugal plates; small triangular tubercles in the maxillary plates; and branching ridges in the nasal and rostral plates. In addition to a superficial layer consisting of odontodes and a basal lamellar bone layer, a middle vascular bone layer is variously developed in the cranial dermal bones.

**Figure 8:**
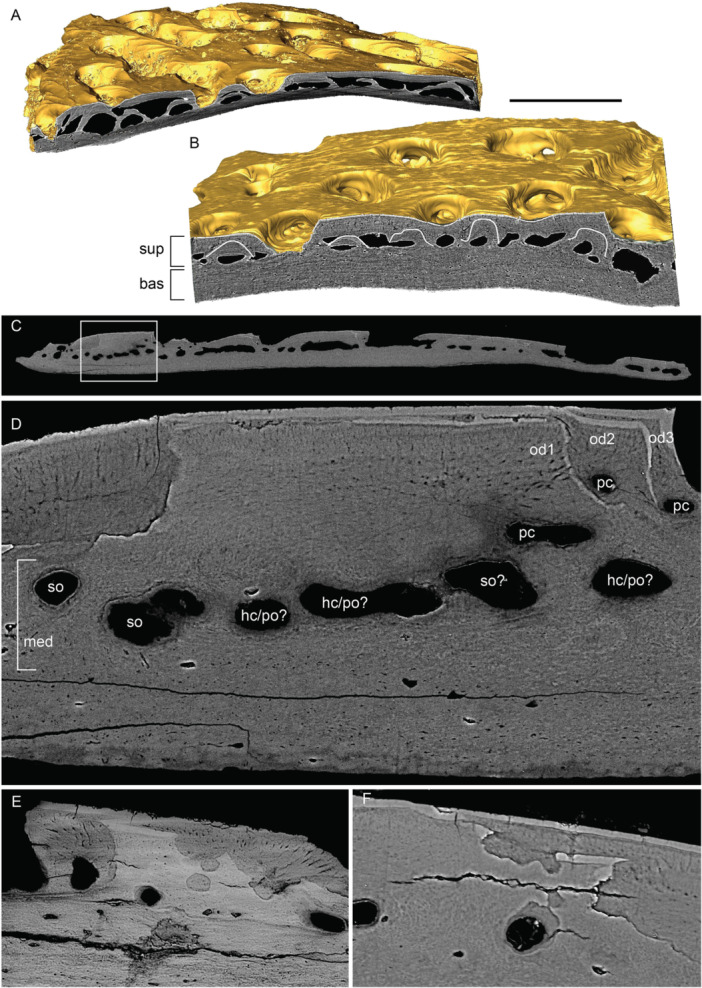
Branchiostegals of *Moythomasia durgaringa*. (A, B) Surface renderings (gold) based on SRXTM data, with a transverse section showing an SRXTM image of a juvenile branchiostegal (A, NHMUK PV P. 56425) and an adult branchiostegal (B, NHMUK PV P.53221). (C–F) SEM images of an adult specimen (NHMUK PV P.53221) in the transverse section. (C) Overview of divisions. (D) Enlargement of (C) showing an area of vascular bone (E, F). Resorption patterns within the superficial layer. bas, basal layer; hc, horizontal canal; med, medial layer; od, odontode; pc, pulp canal; po, primary osteon; and sup, superficial layer. Scale bar = 380 µm (A, B), 1685 µm (C) and 100 µm (D–F).

**Figure 9:**
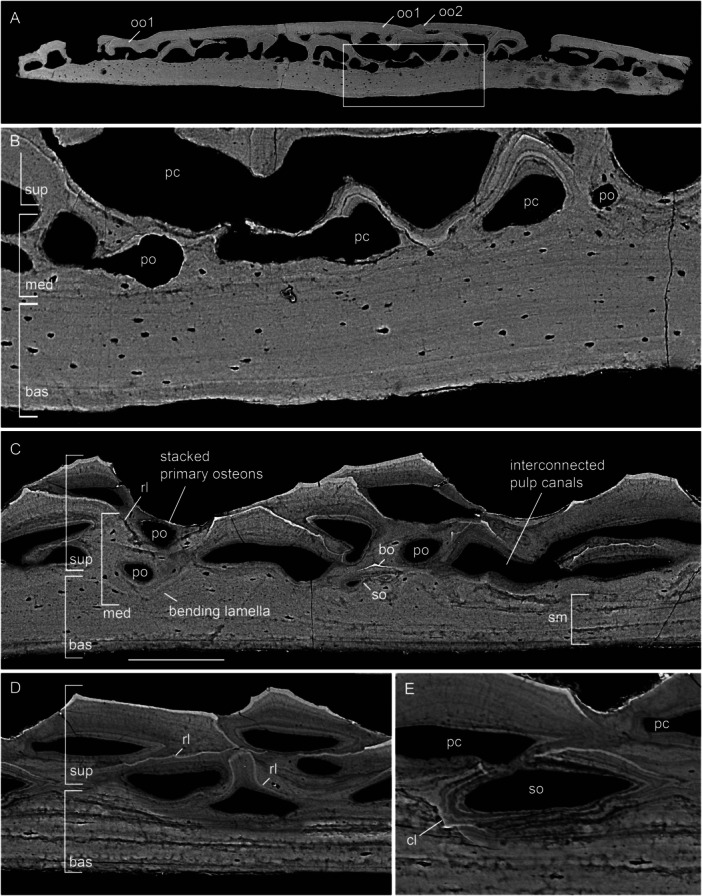
Gular plate of the intermediate developmental stage (A, B) and subopercular (C–E) of *Moythomasia durgaringa*. (A) Overview of composition with an odontode overlay present and multiple pulp canals. (B) Detail showing the middle division (bracket), with osteons present in between odontodes. (C) Overview of the divisions. (D) Basal division solely consisting of spherically mineralized bone. (E) Secondary osteon. bas, basal layer; bo, buried odontode; cl, cementing line; med, medial layer; oo, odontode overlay; pc, pulp canal; po, primary osteon; rl, resorption line; so, secondary osteon; and sup, superficial layer. Scale bar = 633 µm (A), 132 µm (B), 149 µm (C), 113 µm (D) and 72 µm (E).

**Figure 10:**
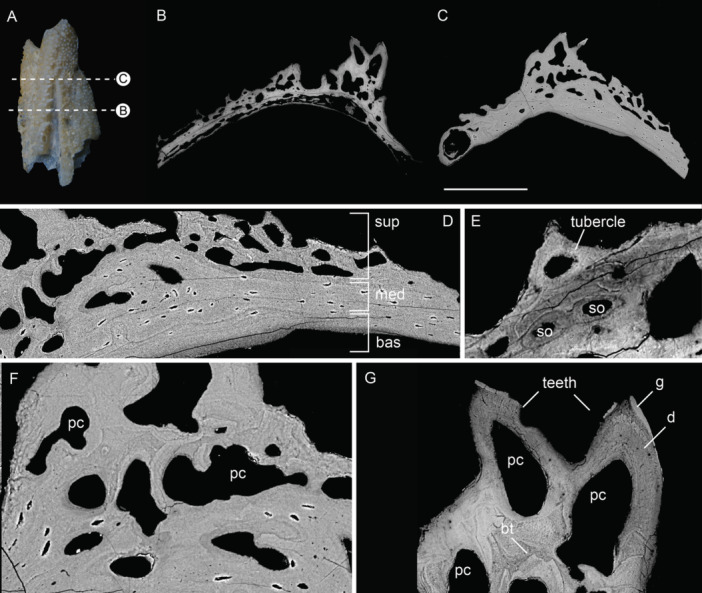
Left juvenile maxillary plate of *Moythomasia durgaringa* (NHMUK PV P.56425). (A) Photo of the element, and direction of the transverse section. (B–C) SEM images showing the structure of the dermal skeleton. (D) SEM images showing basal and middle condensations. (E) SEM images showing detail of tubercle and secondary osteons in the middle division. (F) SEM images showing detail of the tooth row with continuous middle and superficial layers. (G) SEM images showing detail of teeth. bas, basal layer; bt, buried teeth; d, dentine; g, ganoine; med, medial layer; pc, pulp canal; so, secondary osteons; and sup, superficial layer. Scale bar = 1510 µm (A), 422 µm (B), 368 µm (C), 117 µm (D), 50 µm (E), 74 µm (F) and 90 µm (G).

**Figure 11:**
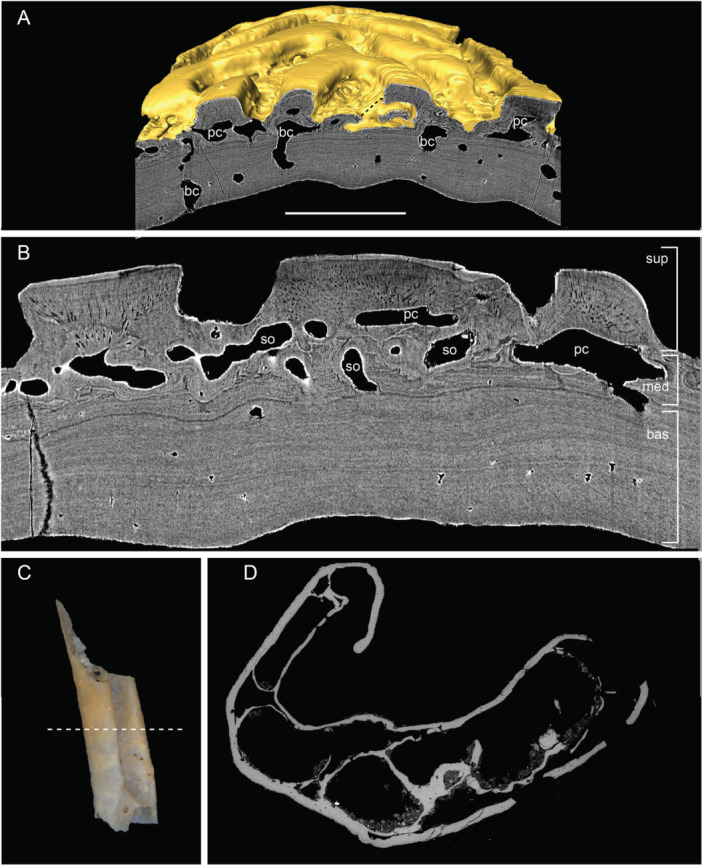
Rostral and hypobranchial plates of *Moythomasia durgaringa*. (A) Surface renderings based on SRXTM data of a rostral plate in an adult specimen (NHMUK PV P.53221), with a transverse section showing the SRXTM image. (B) General composition, with densely packed secondary osteons in the middle division (NHMUK PV P.53221). (C) Photo of the hypobranchial plate and transversal section in dorsal view. (D) Structure of the dermal skeleton showing the absence of the superficial layer. bas, basal layer; bc, basal canal; med, medial layer; pc, pulp canal; so, secondary osteon; and sup, superficial layer. Scale bar = 2295 µm (A), 656 µm (B), 2957 µm (C) and 500 µm (D).

As in the scales, the superficial layer in cranial bones consists of odontodes. However, the shape, number of generations and arrangement of odontodes vary in different cranial elements, leading to the overall difference in external ornamentation. A complete odontode overlay can be observed in the branchiostegal elements of both early and late developmental stages, overlying earlier generations of smaller odontodes (Figure 8A,B). Pulp canals in early‐stage branchiostegal elements are larger than those of later stages (Figure 8A,B). Pulp canals in the gular of intermediate developmental stage are also relatively large, and odontodes often do not completely enclose the pulp canal (Figure 9A,B). The superficial layer of the maxillary consists of irregularly stacked odontodes with presumed teeth that are structurally similar to odontodes of the external dermal skeleton but possess ovoid pulp cavities (Figure 10A–C,G). Buried teeth are partially resorbed and subsequently overgrown by replacement teeth (Figure 10F,G). Tubercles on the surface consist of single odontodes that directly overlie the bone (Figure 10E). The superficial layer of the rostral plate is approximately 175 µm thick, and the external ornamental ridges often consist of a single odontode (Figure 11B) or only very few generations (Figure 11A). An odontogenic superficial layer is completely absent in the hypobranchial plates (Figure 11C,D). Odontogenic resorption is extensive in cranial dermal plates. In the branchiostegal, resorption is progressive, including independent areas of dentine loss, of which some are connected to canals (Figure 8E,F). Details of resorption show an undulating infill of dentine near the resorption area. In general, major differences within the superficial layer lie in the proportion of odontogenic tissue and the number of generations of odontodes.

**Figure 12:**
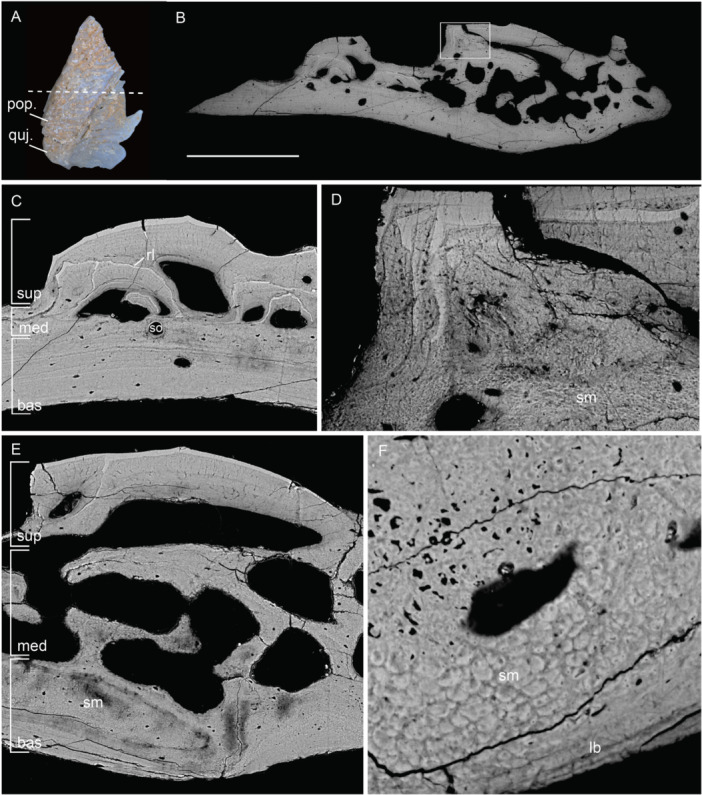
Right preopercular and quadratojugal of *Moythomasia durgaringa* (NHMUK PV P.53219), of the intermediate developmental stage. (A) Photo of the element, and direction of the transverse section. (B) SEM images showing the structure of the dermal skeleton. (C) SEM images showing an overview of the divisions, including a small area with secondary osteons. (D) SEM images showing detail of stacked odontodes in (B) and the detail of spheritic mineralization. (E) SEM images showing a large vascular network within spheritic bone. (F) SEM images showing detail of spheritic mineralization. bas, basal layer; lb, lamellar bone; med, medial layer; pop, preopercular; quj. quadratojugal; rl, resorption line; sm, spheritic mineralization; and sup, superficial layer. Scale bar = 1115 µm (A), 498 µm (B), 200 µm (C), 63 µm (D), 190 µm (E) and 27 µm (F).

A vascularized middle layer is variably present in cranial dermal bones, which directly underlies the superficial layer (Figure 12E) or, in some cases, lies in between stacked odontodes (Figure 9C). This layer consists of cellular bone containing canals that are primary osteons, secondary osteons or horizontal canals connected to the pulp canals. The distribution of fibers in the bone of the middle layer is less organized compared to the strict horizontal distribution in the basal lamellar bone layer. Primary osteons are identified by vascular canals circumscribed by concentric bone lamellae that are centripetally deposited without resorption at the previous periphery of the canal (Francillon‐Vieillot et al. 1990). Growth lines of the underlying basal lamellar tissue bend with the shape of the primary osteons (Figure 9B,C). Secondary osteons are the result of bone remodeling, defined as the resorption and redeposition of bone tissue (Francillon‐Vieillot et al. 1990), and are identifiable as a disruption of the original structure, delimited from the surrounding bone by a cementing line of resorption (Figure 9E). After initial resorption, new concentric lamellar bone is deposited around a vascular channel.

**Figure 13:**
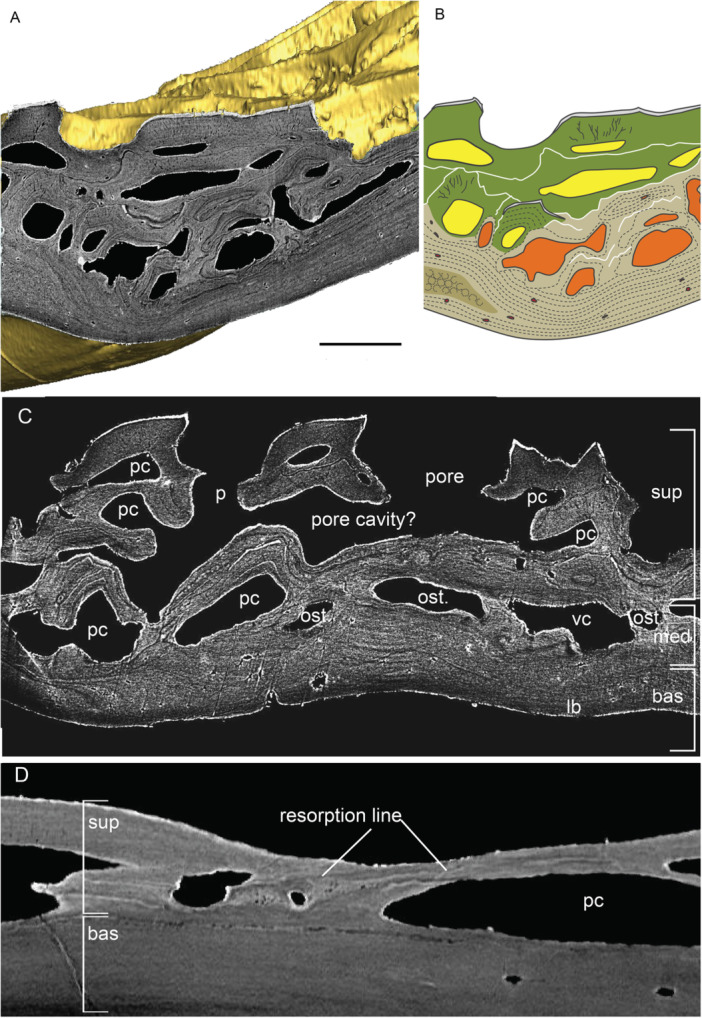
Nasal plates of *Moythomasia durgaringa*. (A) Surface renderings based on SRXTM data of an adult specimen (NHMUK PV P.53221), with a longitudinal section showing an SRXTM image. (B) Schematic model of (A) showing bone (brown), osteocyte spaces (red), spheritic bone (light brown), osteon spaces (orange), dentine + canaliculi (green), pulp canal (yellow), ganoine (gray), arrested growth lines (dashed lines) and resorption lines (white). (C) Pores within the superficial layer (transverse section NHMUK PV P.53221, SRXTM). (D) Detail of the superficial layer of a juvenile specimen showing resorption (NHMUK PV P.56425, SRXTM). bas, basal layer; lb, lamellar bone; med, medial layer; p, pore; pc, pulp cavity; ost., osteon; rl, resorption line; sup, superficial layer; and vc, vascular canal. Scale bar = 100 µm (A), 100 µm (B), 167 µm (C) and 41 µm (D).

This middle layer is clearly distinguishable in the preopercular/quadratojugal (Figure 12E) and nasal (Figure 13A–C), although it varies widely in thickness throughout the bone. In mature branchiostegal, the middle layer is restricted to very limited areas (Figure 8D), whereas in juvenile stages, the middle layer of the branchiostegal is completely absent. In the gular, the middle layer is restricted to the center of the bone, consisting of a narrow strip of less organized bone underlying pulp canals (Figure 9A,B). In the subopercular, primary osteons are not only present in between the oldest generation of odontodes but also younger generations, above resorption lines (Figure 9C). The primary osteons are not isolated within the dentine, but connected to the bone layer, or indirectly via other primary osteons. Growth lines of the underlying basal layer bend with the shape of the primary osteons (Figure 9C), and secondary osteons are also present (Figure 9C,E). In intermediate developmental stages of the preopercular/quadratojugal, the middle layer forms a large division (300 µm in thickness) with an extensive vascular network and a narrow zone of spherically mineralized bone containing secondary osteons (Figure 12B,C,E). In the maxillary, the middle layer is distinguished by a denser distribution of osteocytes, horizontal canals and secondary osteons (Figure 10D); its thickness varies across the element in direct relation to the lamellar bone, and within the tooth row, it occupies a large portion of the structure, continuing into unorganized stacked odontodes with pulp cavities of varying lobate shapes (Figure 10F). In the rostral, the middle layer contains densely distributed secondary osteons (Figure 11B); within elements representing intermediate growth stages, the osteogenic component is on average 50 µm thinner (Figure 11C). Spherically mineralized bone is observed within the middle layer of several elements, such as preopercular and quadratojugal (Figure 12D–F). In general, the middle division varies widely in thickness and composition, consisting of primary and secondary osteons, and horizontal canals that can be connected to openings within the superficial layer.

The basal layer is made up of lamellae deposited in parallel in a horizontal orientation, with the thickness varying among different elements (265 µm in branchiostegal; 110 µm in subopercular; 377 µm in maxillary; 803 µm in rostral; 30 µm in preopercular; 140 µm in nasal). The osteogenic component in juvenile specimens is distinctly thinner than that in the adult, as observed in branchiostegal (40 µm in juvenile, Figure 8A) and nasal (50 µm in juvenile, Figure 13D). Osteocytes in the gular are relatively large and more densely distributed compared to the other cranial elements (Figure 9A,B). A small layer of fine lamellar bone occurs directly beneath the odontodes in the subopercular (Figure 9C), whereas the unornamented part and adjacent area consist of thick, irregular and undulating lamellae, consistent with spheritic mineralization (Figure 9C,D). Areas of spheritic mineralization also occur within the lamellar bone of the preopercular/quadratojugal (Figure 12E,F) and the nasal (Figure 13A,B).

**Figure 14:**
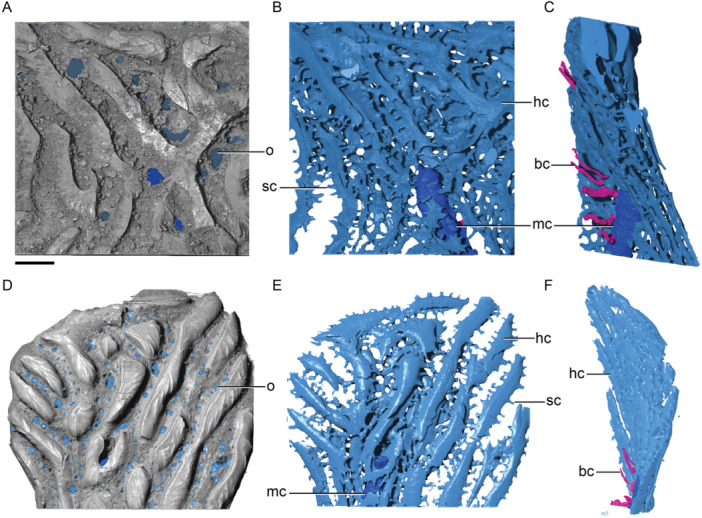
Vascular canal of nasal plates of *Moythomasia durgaringa*. (A, D) Dorsal view of nasal plates in an adult specimen (A) and a juvenile specimen (D), showing numerous surface openings (o) in between ornamental ridges. (B, E) Dorsal view of vascular canals of nasal plates in an adult specimen (B) and a juvenile specimen (E), showing the interconnected horizontal canals (hc, light blue) with side canals (sc, light blue); a main canal (mc, deep blue) is present. (C, F) Lateral view of vascular canals in an adult specimen (C) and a juvenile specimen (F), showing the basal canal (bc, red). Scale bar = 150 µm.

**Figure 15:**
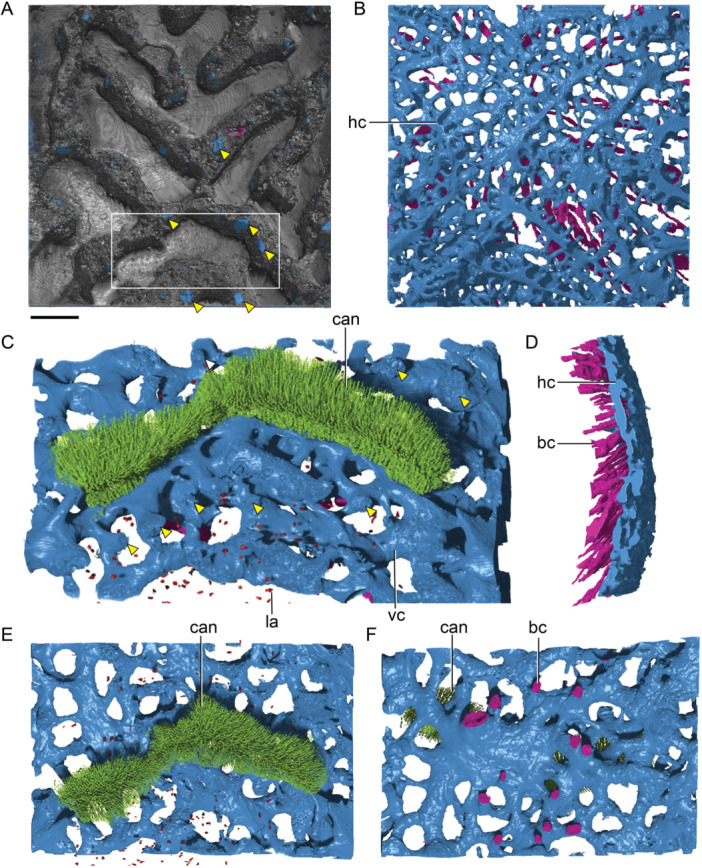
Vascular canal of the rostral plate of *Moythomasia durgaringa*. (A) Dorsal view of the rostral plate in an adult specimen, showing numerous surface openings (arrowheads) in between ornamental ridges. (B, D) Dorsal (B) and lateral (D) views of vascular canals, showing the interconnected horizontal canals (hc, light blue) and basal canals (bc, red); note that a main canal observed in the nasal plate is absent here. (C, E, F) Lateral (C), dorsal (E) and basal (F) views of reconstruction of a single odontode (box region of A) and its underlying vascular canal (vc, light blue), showing the arrangement of canaliculi (can, light green) within the dentine, osteocyte lacuna (la, sparse dots in red). Scale bar = 300 µm.

The canal system present in the adult and juvenile nasal bones, as well as in the adult rostral, was reconstructed from SRXTM data (Figures 14, 15). It can be divided into a set of vertically or obliquely oriented basal canals (bc) embedded within the lamellar bone layer, and a horizontal canal network (hc) comprising vascular canals in the middle layer and pulp canals supplying the dentine (Figure 14). A surface view of the canals shows a regular distribution of pores between ridges, particularly in the juvenile nasal (Figure 14D). These pores vary in size, with regularly distributed small pores representing openings of side canals (sc) branching from horizontal pulp canals (hc), whereas larger pores represent openings of ascending vascular canals from the middle or basal layers. A main channel (mc) is present in both juvenile and adult nasals (Figure 14B,C,E,F) but absent from the rostral (Figure 15B,D), which instead shows a greater number of basal canals than the nasal (Figure 15D). In the juvenile nasal, the horizontal canals (hc) extend longitudinally throughout the base of odontodes, connected by small transverse side canals or across canals within the middle layer. In the adult nasal, an increase is seen in the number of generations (representing buried odontodes), with a change from neatly organized channels to a disorganized, interconnected network (Figure 14A–C). Older channels of buried odontodes remain connected to the network. A number of canals originating from the middle layer ascend towards the superficial layer and open to the bone surface (Figure 15C,E). These ascending canals supply the horizontal pulp canals that supply the dentine (Figure 15C,E,F), indicating a close relationship of odontode addition and the development of vascular bone within the middle layer.

**Figure 16:**
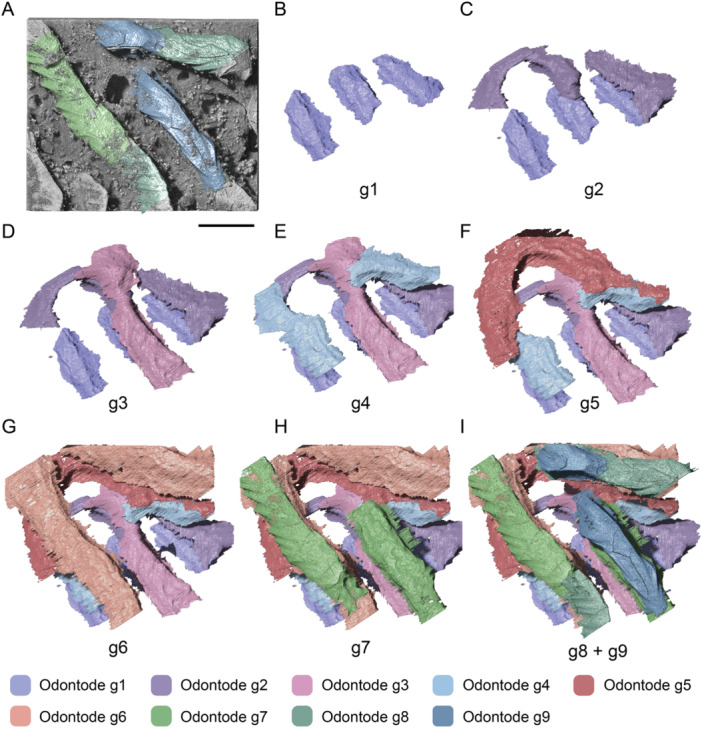
Reconstruction of the growth of the nasal plate of *Moythomasia durgaringa*. (A) Part of the nasal plate in an adult specimen. (B–I) Gradual addition of odontode generations 1–9 (g1–g9). Scale bar = 300 µm.

The hypermineralized layers and associated resorption surfaces in part of the nasal plates were reconstructed to show the growth history of the odontodes. Nine generations of odontodes (g1–g9) are identified, with the last three generations contributing to the surface of the ornamental ridges (Figure 16A). Odontode g1 (Figure 16B) consists of three independent elongated ridge‐like odontodes with similar length and grows deep inside the middle vascular bone. The odontode ridges are nearly parallel with each other. Odontode g2 contains a U‐shaped odontode that partially overlaps g1 (Figure 16C). U‐shaped odontode can also be observed in generation 5 (g5, Figure 16F). Generally, odontodes g2–g9 (Figure 16C–I) are added appositionally, with each new generation partially or completely overlapping the previous one, increasing the thickness of the dermal bone.

### 3.4 Ancestral State Estimation of Osteichthyan Skeletal Characters

The histology of the dermal skeleton of *Moythomasia durgaringa* is now among the best characterized of any early osteichthyan and so we consider what is known of the dermal skeleton of other osteichthyans in its light. For the two osteichthyan crown clades, the principal distinction in the composition of their dermal skeleton is the presence of ganoine (multi‐layered enamel) in actinopterygians and cosmine (a dermal complex of hard tissue and vascular systems) in sarcopterygians (e.g., Sire et al. 2009). However, the discovery of cosmine‐like characters in early osteichthyans (*Meemannia*, *Cheirolepis* and *Psarolepis*) has raised the question of whether cosmine is a synapomorphy of sarcopterygians or a synapomorphy of osteichthyans (Lu et al. 2016). To investigate the phylogenetic distribution of cosmine and clarify the histological nature of the ancestral osteichthyan, we collected data on cosmine‐related characters from early vertebrate taxa spanning the phylogenetic diversity of extinct jawed vertebrates.

We conducted a multiplicative set of ancestral states analyses, accounting for (1) different phylogenetic hypotheses and (2) different data sets from cranial and postcranial dermal skeletons. Three tree topologies were selected in this analysis. One tree topology is obtained from Qiao et al. (2016), in which *Guiyu*, *Achoania* and *Psarolepis* (GAP clade) are resolved as stem‐Sarcopterygii, and the other two tree topologies are from King et al. (2017), in which the GAP clade is resolved as stem‐Osteichthyes and stem‐Actinopterygii, respectively. We time‐scaled three trees using the function *timePaleoPhy* in the R package (https://www.R‐project.org/) *paleotree* (Bapst 2012) using the “equal” method, with the root age increased by 5 million years.

As cosmine is a dermal complex of hard tissue and vascular systems, with debate on the structure and development of these tissues, we coded each taxon for five characters: (1) dermal odontode skeleton: absent, present; (2) enamel: absent, present; (3) enamel layer: single‐layered; multi‐layered; (4) odontogenic resorption: absent, present; and (5) vascular canals: one dentinal canal system, an additional rudimentary pore canal system, which describe dentinal canals supplied by horizontal canals embedded in bone with irregular pore cavities and openings, as observed in *Moythomasia*, and an additional typical pore canal system, describing regularly patterned mesh canal system with flask‐shaped pore cavities and openings. Here, we use the term dermal odontode skeleton to refer to dermal elements that contain one or more odontogenic tissues, including dentine, enamel or enameloid, and bone of attachment. As cosmine is a tissue complex occurring exclusively in the external dermal skeleton, characters associated with teeth are not considered in this analysis. Dermal enamel and ganoin are considered homologous in our analysis, and ganoin described in some taxa are coded as muti‐layered enamel. In our analysis, characters are hierarchically related. The states of characters 2, 3, 4, and 5 are dependent on character 1 (dermal odontode skeleton, state = present) and the state of character 3 (enamel layer) is dependent on character 2 (enamel, state = present). All characters were amalgamated into a single structured Markov model (SMM) equipped with hidden states, which is demonstrated to be able to resolve the problem of modeling character complexes with hierarchical dependencies (Tarasov 2019, 2023). We amalgamated all characters into a single SMM with 25 states. Ancestral states were estimated under the SMM_sw model, which assumes that character 2 (enamel) can only change state if character 1 (dermal skeleton) is in a specific state (state = presence).

**Figure 17:**
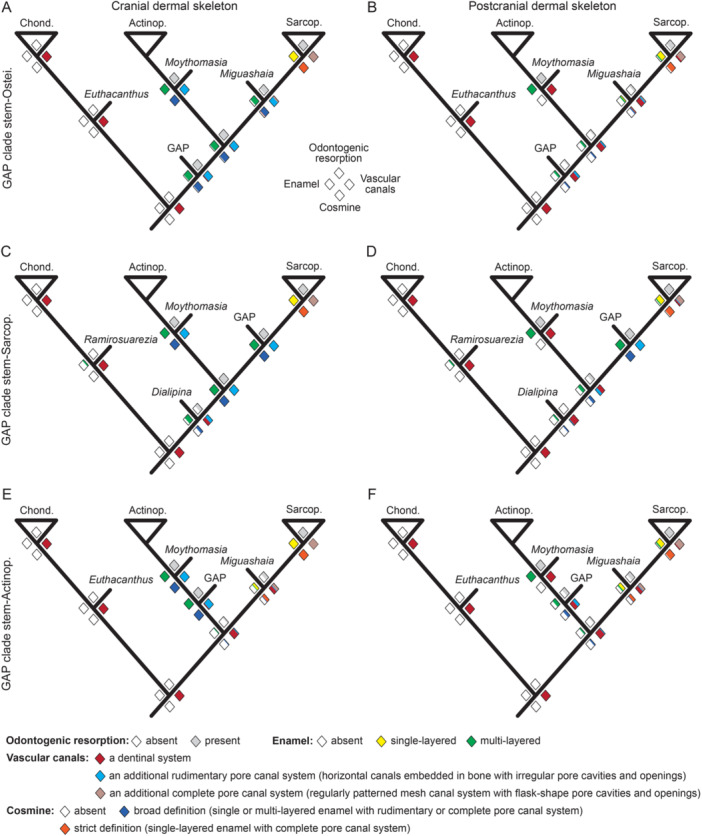
Estimation of ancestral states of dermal skeleton tissues and characters. (A–F) Ancestral state estimation of the forms of cosmine and its associated characters in the cranial (A, C, and E) and postcranial (B, D, and F) dermal skeletons for trees, in which the GAP clade is resolved as stem‐Osteichthyes (A and B), stem‐Sarcopterygii (C and D) or stem‐Actinopterygii (E and F). Diamonds at nodes express the probability of the state of a specific character or character complex, with the proportion of the color fill reflecting probability. The crown‐chondrichthyans (Chond.), crown‐Actinopterygii (Actinop.) and crown‐Sarcopterygii (Sarcop.) are shown as triangles, and important stem taxa are shown by bars (e.g., *Moythomasia*). More detailed results are presented in Supporting Information S1: Figures 1–24. Supporting Information S1: Tables 1–4 show data used to construct these trees.

Our estimates of ancestral states show variations under different phylogenetic placements of the GAP clade (Figure 17; Supporting Information S1: Figures 1–24). The evolutionary patterns are broadly similar when the GAP clade is resolved as either stem‐Sarcopterygii or stem‐Osteichthyes (Figure 17A–D). The results indicate that dermal enamel evolved deep within Osteichthyes stem lineages (*Dialipina* or GAP clade) in the form of multiple layers, supporting the hypothesis inferred from molecular data that an ancestral enamel was present at the osteichthyan crown‐group node (Qu et al. 2015; Kawasaki et al. 2021). Multi‐layered enamel is retained in both stem‐actinopterygians (*Moythomasia*) and stem‐sarcopterygians (GAP clade or *Miguashaia*), whereas it became single‐layered in crown‐sarcopterygians. Odontogenic resorption is inferred to be ancestral for crown‐osteichthyans, and it is retained in stem‐actinopterygians, stem‐sarcopterygians and early crown‐sarcopterygians, including the common ancestor of *Powichthys*, *Youngolepis* and *Diabolepis* (Supporting Information S1: Figures 13–18), contrary to some previous suggestions (Schultze 2016). Regarding vascular canals, the cranial and postcranial dermal skeleton shows a different evolutionary pattern. A dentinal system is shared by chondrichthyans and osteichthyans. On this basis, a rudimentary pore canal system, comprising horizontal canals embedded in bone and opening in between odontodes, evolved in the cranial dermal skeleton of stem‐osteichthyans and was retained in stem lineages of both actinopterygians and sarcopterygians (Figure 17A,C). However, it is inferred to be absent from the postcranial dermal skeleton outside the sarcopterygian crown‐group (*Miguashaia* or more crownward, (Figure 17B,D). An additional regularly patterned mesh canal system is absent for ancestral crown‐osteichthyans, having evolved in crown‐sarcopterygians in both the cranial and postcranial dermal skeletons, although it evolved convergently in osteostracans and acanthodians (Supporting Information S1: Figures 7–12). In a broad sense, cosmine, composed of enamel associated with a rudimentary pore canal system, appeared in the cranial dermal skeleton of crown‐osteichthyans (Figure 17A; Supporting Information S1: Figures 19, 21), but it was absent from the postcranial dermal skeleton (Figure 17B; Supporting Information S1: Figures 22, 24). True cosmine, defined by a single layer of enamel combined with a regularly patterned pore canal system, evolved exclusively in crown‐sarcopterygians in both cranial and postcranial dermal skeletons (Figure 17A–D).

Our estimates of ancestral states show different results when the GAP clade is resolved as stem‐actinopterygians (Figure 17E,F). In this condition, enamel and odontogenic resorption is inferred to be absent in the crown‐osteichthyans and evolved independently in actinopterygians and sarcopterygians. However, this might be caused by the exclusion of osteichthyan stem taxa (i.e., *Andreolepis*) in this selected tree topology. Nevertheless, the evolution of vascular canals in the cranial and postcranial dermal skeleton also differs, particularly in actinopterygians. Cosmine in a broad definition is never present in the postcranial dermal skeleton of actinopterygians and true cosmine evolved exclusively in crown‐sarcopterygians (Figure 17E,F).

## 4 Discussion

### 4.1 Variation Within the Dermal Skeleton of *Moythomasia durgaringa*

All elements of the dermal skeleton of *Moythomasia* consist of a superficial layer of stacked odontodes and an inner layer of lamellar bone. A middle layer is variably present, absent in scales, limited in bones of the branchiostegal apparatus and widespread in cranial dermal bones. The superficial layer varies in external ornamentation and subsequent compositional differences (distribution of odonto‐complexes along the element and organization of stacked odontodes), the number of generations and presence of pores with interconnecting canals. The thickness and composition of the middle layer vary, from making up the majority of the element to a very narrow condensation. It can consist of either vascular bone with secondary osteons and horizontal canals, primary osteons present in between odontodes, bone with randomly organized fibers, areas of spheritic mineralization and combinations of these. The basal layer is shared by all the elements. Evidence for spheritic mineralization occurs only in cranial dermal bones of *Moythomasia*; it is absent from scales. In terms of the growth pattern of odontodes, the dermal scales of *Moythomasia* display a polarized growth pattern that is not observed in the cranial dermal bones.

Variation in the histological composition of the dermal skeleton of *Moythomasia* across the trunk and cranium is common in extant actinopterygians, in which cranial dermal bones show a tripartite structure and the scales are variously elasmoid or ganoid (Sire et al. 2009). Such head–trunk skeletal differentiation has a phylogenetically early origin, as seen in heterostracans and osteostracans, where the middle vascular layer is well developed in the headshield but reduced or completely absent in the trunk scales (Keating et al. 2015; O'Shea et al. 2019). This is an important point, as most histological studies of the dermal skeleton focus solely on scales.

### 4.2 Growth and Development in *Moythomasia durgaringa*

Comparison of early and late developmental stages (scale and nasal bone) shows substantial growth of both the odontogenic and the osteogenic component throughout ontogeny, accompanied by an increase in the size of odontodes. Interstitial growth of dentine infilling pulp canals continues throughout the ontogeny, inferred from the decreasing size of pulp canals and increasing amount of dentine in the successive stages. The stacked odontodes represent different generations; in between, resorption lines are visible affecting ganoine and underlying dentine. Within the superficial layer of scales, odontodes add along two polarities and those of one generation never completely cover the previous generation, conforming to a polarized areal growth pattern, whereas in the cranial bones, such as the nasal, appositional growth is dominant in the superficial layer. Partial odontogenic resorption is associated with deposition of overlying generations of odontodes and is not a secondary change later in ontogeny. New generations of odontodes are preceded by partial resorption of the pre‐existing layer, likely to have affected the whole surface, including the osteogenic components.

The middle division is absent in early ontogenic stages and expands interstitial towards the superficial layer. The middle layer displays a large developmental difference among the dermal bones. This is related to differential changes in shape through ontogeny (such as in the nasal bone and preopercular/quadratojugal).

The lamellar bone in the basal layer grows in an appositional pattern with a new bony tissue layer added basally. Spherically ossified bone is observed in the basal layer of cranial bones (nasal, gular, preopercular and quadratojugal), reflecting rapid growth (Downs and Donoghue 2009). Resorption of the lamellar bone is extensive, particularly in the scales. As scales grew, the two ossification centers became more widely spaced, gradually migrating away from one another. As the scale peg grows larger, the socket region undergoes extensive resorption to expand its space to accommodate the peg of its adjacent scale neighbor. This mechanism of growth allows enlargement while maintaining function.

The vascular network consists of longitudinal canals running through the odontodes in multiple generations, interconnecting all canals within the elements. Secondary osteons belonging to this network are limited to the medial division and show that *Moythomasia* had the ability to remodel bone, and therefore, the possibility of repair.

### 4.3 Comparison to Other Early Vertebrate Taxa

The cranial dermal skeleton of stem‐actinopterygian *Moythomasia durgaringa* consists of three layers: a superficial layer composed of multi‐layered dentine capped by ganoine; a vascular middle layer consisting of cellular bone; and a basal layer composed of cellular lamellar bone. A tripartite division of the dermal skeleton, comprising an odontogenic component forming the superficial layer and an osteogenic component forming the middle and basal layers, has been present since the earliest occurrence of dermal skeleton, although it initially consists of acellular bone (Keating et al. 2015). The organizational pattern in which odontodes (odontogenic tissue) are supplied by a vascular canal network enclosed within osteogenic tissue is also observed in heterostracans and osteostracans (Keating et al. 2015; O'Shea et al. 2019) and is widely present in early osteichthyans (Gross 1956; Qu et al. 2017). Bone remodeling is widespread in the cranial bones of *Moythomasia*, and it has also been observed in stem‐gnathostomes heterostracans, osteostracans and placoderms (Donoghue and Sansom 2002; Donoghue et al. 2006b; Giles et al. 2013; Keating et al. 2015; O'Shea et al. 2019). Spheritic mineralization has been reported to widely occur throughout the stem‐gnathostomes (Downs and Donoghue 2009), as a common characteristic of the vertebrate dermal skeleton.

The histology of the postcranial dermal skeleton of stem‐actinopterygian *Cheirolepis* is well described, consisting of a bony basal plate covered by dentine and ganoine (Zylberberg et al. 2015), comparable to the scale composition of *Moythomasia*. In both taxa, superimposed ganoine layers are laterally separated by dentine, and each odontode is organized around a vascular canal, showing partial odontogenic resorption (Zylberberg et al. 2015). In *Moythomasia*, numerous pores occur on the scale surface, either at the bases of odontodes or penetrating the ganoine, whereas in *Cheirolepis,* the scale surface is covered by extensive ganoine without pores. Moreover, the scales of *Moythomasia* differ from those of *Cheirolepis* in having multiple odontodes within a single generation, whereas only a single odontode develops for each generation in *Cheirolepis*. In stem‐actinopterygians, more crownward than *Moythomasia*, similar features of the dermal skeleton are present, including stacked odontodes, occasional pores, bone remodeling and odontogenic resorption (e.g., *Birgeria* and the palaeonisciform *Scanilepis*, Ørvig 1978). This suggests that the condition in *Moythomasia* reflects a generalized actinopterygian condition. In terms of the scale histology, a wide variety of scale types have been recognized in actinopterygians: palaeoniscoid scales (stem‐actinopterygians), polypteroid scales (polypteriforms), lepidosteoid scales (lepisosteiforms) and elasmoid scales (teleosts). Within the palaeoniscoid scale, tissue types similar to *Moythomasia* occur. The odontocomplex (superposition of odontodes) occurs also in more crownward stem‐actinopterygians such as *Scanilepis* (Ørvig 1978) and crown‐polypteroids; it should be considered a symplesiomorphy of actinopterygians. Scales in extant *Polypterus* differ from *Moythomasia* scales in the presence of an additional “elasmodine” tissue underneath the superficial condensation. In most living actinopterygians and groups within Sarcopterygii, elasmodine is present as the characterizing part of the elasmoid scale. The widespread occurrence of the elasmoid scale (and similarity to early development of polypteroid scales) led Sire et al. (2009) to conclude that the elasmodine is present at least in the last common ancestor of osteichthyans and represents a very ancient vertebrate tissue. The absence of elasmodine in *Moythomasia* as well as in other early osteichthyans (e.g., *Andreolepis*, *Lophosteus*, and *Psarolepis*, Qu et al. 2013a, 2013b) is not compatible with this hypothesis.

Several late Silurian–Early Devonian taxa (*Andreolepis*, *Dialipina*, *Lophosteus*, *Ligulalepis*, *Meemannia* and *Psarolepis*) have been variably resolved as stem‐osteichthyans, stem‐actinopterygians or stem‐sarcopterygians, helping to clarify the phylogenetic distribution of skeletal characters. Among them, *Andreolepis* and *Lophosteus* have consistently been resolved as stem‐osteichthyans (Zhu et al. 2009; Dupret et al. 2014; Long et al. 2015), whereas the others have been variously recovered in all three positions, with little consensus regarding their relationships (Zhu et al. 2009; Dupret et al. 2014; Long et al. 2015; King et al. 2017; Lu et al. 2017; Cui et al. 2025). A comparison of the histological condition in *Moythomasia* and these taxa reveals a large number of similarities, including the presence of a three‐layered dermal skeleton, stacked odontodes, odontogenic resorption and bone remodeling.

The cranial dermal skeleton of *Moythomasia* contains a middle layer of cellular vascular bone, which is variably developed (not uniformly distributed) and most pronounced in regions where the dermal bone is thick. A similar condition is also present in the cranial dermal bones of *Andreolepis* (Cunningham et al. 2012; Chen et al. 2016), *Meemannia* (Zhu et al. 2010), *Psarolepis* (Qu et al. 2013a), *Lophosteus* (Gross 1968; Chen et al. 2020) and *Ligulalepis* (Burrow et al. 2023), indicating that variation in this middle division is a symplesiomorphy of osteichthyans. The vascular structure related to the middle layer and the superficial odontode layer in stem‐ and early crown‐osteichthyans has been the subject of debate. In particular, the pore canal system that characterizes cosmine has been interpreted to be present in the cranial dermal bones of *Psarolepis*, *Meemannia* and *Cheirolepis* (Zhu et al. 2010; Lu et al. 2016), and along with the placement of *Meemannia* as a stem‐actinopterygian, a pore canal system is considered to be a symplesiomorphy of osteichthyans (Lu et al. 2016). From a two‐dimensional histological perspective, the cranial bones of *Moythomasia* have horizontal canals lying at the base of the oldest odontodes and connecting directly with adjacent dentinal pulp cavities or canals. Histological sections of cranial bones in *Psarolepis*, *Meemannia* and *Cheirolepis* show a similar condition, in which the horizontal canals have a direct connection to the pulp cavities sometimes (Zhu et al. 2010; Qu et al. 2013b). Although the three‐dimensional network of the vascular canals in *Psarolepis* and *Cheirolepis* is geometrically regular, reminiscent of a pore canal system (Lu et al. 2016), this structure represents only part of the vascular canal system, and its relationship to the pulp cavities supplying to odontodes remains unclear. This relationship is critical for assessing homology among vascular structures, because in rhipidistian sarcopterygians, the pore canal system is clearly separated from the pulp cavities. Our data from *Moythomasia* provide a model for the vascular network of dermal elements that appear to possess a pore canal system in two‐dimensional histological sections. The vascular network in the cranial bones of *Moythomasia* is far less organized, lacking a regular geometric arrangement of horizontal canals, flask‐shaped cavities and, particularly, a clear distinction between horizontal canals and pulp cavities. This contrasts with the pore canal system of porolepiforms, dipnoans and osteolepiforms, in which horizontal canals form a mesh canal network that never directly connects with the canals supplying the odontodes. Qu et al. (2017) described a less regular pore canal system in scales of *Psarolepis* composed of incompletely connected horizontal canals that shows a clear distinction to the other vascular canals. On this basis, they proposed a stepwise evolution of the true pore canal system of sarcopterygians from such an irregular condition. However, the interpretation of the pore canal system in *Psarolepis*, *Meemannia* and *Cheirolepis* has been questioned by Schultze (2018). Our three‐dimensional data from *Moythomasia* do not support the proposed homology between the so‐called pore canal system in these taxa and that of sarcopterygians, highlighting the need for more three‐dimensional histological data on the complete vascular structure of both the cranial and postcranial dermal skeletons of stem‐ and early crown‐osteichthyans.

The organization of the canal system in scales of *Moythomasia* is comparable to that of *Lophosteus* and *Andreolepis*, consisting of slightly tilted canals in the basal layer and a horizontal canal network in the superficial layer. There are two basal canals connected with a horizontal vascular network in scales of *Andreolepis*, three in *Lophosteus* and two or three in *Psarolepis* (Qu et al. 2013a, 2017; Jerve et al. 2016). By contrast, the basal layer of the *Moythomasia* scale that we studied contains up to 17 canals connected with the superficial horizontal canals. These basal canals show a diffuse, multiple‐entry–point relationship to the surficial vasculature, as they underlie odontodes of different generations. This relationship is not found in the scales of *Lophosteus*, *Andreolepis* or *Psarolepis*.

Another important characteristic of cosmine is complete resorption before a new generation of odontodes is deposited, to free sutures and facilitate growth (Thomson 1977). More generally, odontogenic resorption is widespread in stem‐ and early crown‐osteichthyans and likely originated early in stem‐gnathostomes. Resorption associated with teeth has been observed in stem‐osteichthyans *Lophosteus* and *Andreolepis,* in which teeth can be shed and replaced by basal resorption (Chen et al. 2016, 2020). Resorption surfaces related to teeth have also been reported on gnathal elements of arthrodires such as *Eastmanosteus* and *Bullerichthys* (Johanson and Smith 2005; Johanson and Trinajstic 2014; Trinajstic et al. 2025). In stem‐gnathostome, an exception of resorption linked to odontodes of the superficial layer on cranial bones is related to sensory line growth in *Romundina gagnieri* (Olive et al. 2025). In *Moythomasia*, the partial odontogenic resorption is extensive in both cranial and postcranial dermal skeletons; a similar condition is also observed in the dermal odontode skeleton of *Psarolepis* and basal sarcopterygians *Styloichthys* (Cui et al. 2025). In contrast, *Andreolepis* shows odontogenic resorption on cranial bones but lacks resorption on trunk scales, whereas *Lophosteus* lacks resorption entirely in its dermal odontode skeleton, except in the teeth (Chen et al. 2016, 2017, 2020).

Unlike *Moythomasia*, the dermal skull of *Meemannia* reportedly shows no evidence of odontogenic tissue resorption (Zhu et al. 2010). Complete odontogenic resorption is argued to be associated with cosmine in sarcopterygians, such as *Ectosteorhachis* and *Megalichthys*, in which periodic complete resorption of odontogenic tissues is interpreted as necessary for the growth of cosmine (Thomson 1975, 1977; Meinke 1982, 1984; Borgen 1989). Zhu et al. (2010) placed *Meemannia* together with *Andreolepis* and actinopterygians as a group without resorption at the base of their cladogram, whereas Schultze (2016) argues that partial resorption is present in *Meemannia*. Considering the presence of odontogenic resorption in the cranial bones of stem‐actinopterygians *Moythomasia* and stem‐osteichthyans *Andreolepis*, the reported absence of odontogenic resorption in the cranial bones of *Meemannia* requires further confirmation using 3D histological data, as this feature is difficult to verify based on 2D sections.

Odontogenic resorption is present in both sister groups of osteichthyans but differs in some respects. In sarcopterygians, odontode generations are almost always separated by bone (e.g., *Styloichthys*, *Griphognathus* and *Spermatodus*), which is not the case in cranial bones of *Moythomasia*, with only limited bone between generations of odontodes. In more derived actinopterygian taxa, both forms of structures can be seen, also combined (Ørvig 1978). A strict distinction is the apparent absence of resorption surfaces or lines in sarcopterygians, in which odontode generations and surrounding bone form a continuous depositional structure. Likely related to this, the occurrence of resorption at the bottom of odontodes was only observed in sarcopterygians. It resembles the early ontogenetic stages of cosmine with resorption, creating resorption spaces at the base of the cosmine. Cosmine is furthermore uniquely characterized by the completeness of resorption, affecting both odontogenic and osteogenic tissues without following internal architecture. Thomson (1975) therefore suggested that the cells involved are unlike resorption cells in extant taxa. Resorption in actinopterygians serves to prepare the surface for a new generation, whereas in sarcopterygians, resorption is concentrated on replacing odontogenic tissues with bone matrix.

The formation and organization of polyodontode scales of *Lophosteus*, *Andreolepis* and *Psarolepis* have been well investigated using microtomography (Qu et al. 2013a, 2013b, 2017; Jerve et al. 2016), which enables a comparison of the growth pattern of odontogenic tissues. The scales of *Moythomasia* are ornamented with ridges constructed by odontodes, similar to the scales of *Lophosteus* and *Andreolepis*, but differing from the scales of *Psarolepis*, which have a smooth, cosmine‐like surface. In the scales of *Andreolepis* and *Psarolepis*, each generation consists of a single odontode, and successive generations of odontodes can be distinguished by their overlapping relationships. This is different from *Lophosteus* and *Moythomasia* scales, in which each generation comprises multiple odontodes that are spatially separated from each other. These separated odontodes are grouped into a single generation because their relative sequence of addition cannot be determined. In *Lophosteus*, odontodes of each generation are in contact with the underlying odontodes of the previous generation by a bone of attachment, rather than through direct dentine‐on‐dentine contact (Jerve et al. 2016). This contrasts with the pattern in scales of *Andreolepis*, *Psarolepis* and *Moythomasia*, in which odontodes in two successive generations are in direct dentine‐on‐dentine contact, comprising odontocomplexes. The scale crown of *Lophosteus* is composed of four generations of odontodes; younger generations are added in a gap‐filling pattern, although it will partially overlap previous generations. In *Andreolepis*, odontodes 1 to 5, which grouped into the first generation, show a highly organized sequential addition, with each younger odontode being added next to the dorsal older one and partly overlapping it (Qu et al. 2013a). This is similar to the polarized growth pattern observed within a single ridge of the scale of *Moythomasia*. The subsequent generations of odontodes in *Andreolepis* show a gap‐filling pattern with considerable morphological variability, which is similar to the late growth stages of the scale of *Moythomasia* (g10, Figure 7). In contrast to the scales of *Andreolepis* and *Moythomasia*, the scales of *Psarolepis* have a cosmine‐like texture with a smooth enamel cover pierced by surface pores (Qu et al. 2017). In *Psarolepis*, the scale crown comprises seven odontodes that are added in a non‐polarized random pattern. Younger odontodes are added peripherally as patches, leading to the loss of a ridge‐like ornamentation of the scales (Qu et al. 2017). It is noteworthy that both scale odontodes of *Psarolepis* and *Moythomasia* show a cosmine‐like enamel cover with pores (odontode 2 in *Psarolepis* and odontode g9 in *Moythomasia*) that is not observed in scales of *Lophosteus* and *Andreolepis*. The ability to produce cosmine‐like odontodes with surface pores may have evolved in the dermal skeleton of stem‐osteichthyans and maintained in both stem‐sarcopterygians and stem‐actinopterygians.

### 4.4 Implications for the Ancestral Osteichthyan Condition

Comparison of the stem‐actinopterygian condition along the vertebrate lineages provides valuable new insights into the acquisition of characters leading up to the osteichthyan divergence. The dermal skeleton of stem‐ and early crown‐osteichthyans has a cosmine‐like structure associated with a rudimentary pore canal system in at least some of the dermal elements. The results of our ancestral state estimation show that characters related to cosmine have a very sparse distribution in early osteichthyans. Our ancestral state estimation supports an origin of odontogenic tissue resorption within stem‐osteichthyans, retained in both actinopterygians and sarcopterygians, suggesting a much earlier gradual evolution of the characters that culminate in cosmine in crown‐sarcopterygians (Qu et al. 2017).

The pore canal system has been considered to have originated before the divergence of crown‐osteichthyans and so it has been regarded as a synapomorphy of Osteichthyes (Lu et al. 2016). The results of our ancestral state estimation suggest that the pore canal system associated with cosmine is a specialized arrangement of vascular canals that is otherwise present in most dermal skeletonizing vertebrates but associated with an additional mesh canal system. Two independent origins of a pore canal system occur in osteostracans and acanthodians, supporting the independent evolution of the mesh canal before crown‐osteichthyan divergence (Ørvig 1969); its absence from chondrichthyans may merely reflect their reduced dermal skeleton, which is composed of little more than odontodes. The vascular canal system described in *Meemannia*, *Psarolepis* and *Cheirolepis* is similar to that of *Moythomasia*, consisting of horizontal canals connected with adjacent dentinal pulp cavities. More detailed assessments of the 3D structure of canals in these taxa and their relationship to odontode development need to be undertaken to better understand the distribution of cosmine characters and assess the homology to the pore canal system in sarcopterygians. Nevertheless, based on the data that are currently available, it is already clear that cosmine is composed of a complex of characters, and the results of our ancestral state estimation analyses demonstrate that subsets of these are present in groups outside of Rhipidistia in which cosmine was first described and defined (Williamson 1849). At present, there is no good evidence for cosmine existing outside of Rhipidistia but further clarification is needed on the three‐dimensional structure of cosmine in Rhipidistia and the nature of the dermal skeleton in stem‐ and early crown‐osteichthyans.

Based on the comparative analysis and ancestral state estimation, the dermal skeleton of the ancestral crown‐osteichthyan shows variation within cranial and postcranial parts, as observed in the dermal skeleton of *Moythomasia*. The cranial dermal skeleton consists of multi‐layered enamel‐associated horizontal vascular canals embedded in bone and supplying dentinal pulp canals. This represents a composition of the odontocomplex layer and underlying vascular bone, similar to the condition in some cranial dermal elements of *Moythomasia*. In contrast, the postcranial dermal skeleton of the ancestral crown‐osteichthyan lacks such a horizontal canal network associated with the lack of a middle vascular bone layer.

## 5 Conclusions

The dermal skeleton of stem‐ and early crown‐osteichthyans is poorly understood and known mainly from isolated trunk scales, making it difficult to relate to tetrapods that lack a trunk dermal skeleton. We investigate stem‐actinopterygians *Moythomasia* as a model for comparison because it is exceptionally preserved and abundant, allowing us to analyze topological and ontogenetic variations in the structure of the dermal skeleton. Our SEM and SRXTM data reveal that the dermal skeleton of *Moythomasia* comprises a superficial layer of stacked odontodes and a basal layer of lamellar bone, with histological differentiation occurring between dermal elements, particularly in a variously developed middle vascular layer. The high‐resolution synchrotron tomography data enable us to characterize the growth of odontodes within scales and cranial bones, which reveals a different growth pattern. Additionally, we reconstruct the vascular canals of cranial bones of *Moythomasia*, which have horizontal canals lying at the base of the odontodes that supply adjacent dentinal pulp canals and open through pores between odontode ridges, but lack a regularly patterned mesh canal system that characterize cosmine. Finally, ancestral state estimation reveals that characters associated with cosmine are sparsely distributed among early osteichthyans, and cosmine described from Rhipidistia is absent outside this group. These findings suggest a much earlier gradual evolution of cosmine, highlighting the need for further clarification of this character complex, on the three‐dimensional structure of cosmine in Rhipidistia and the nature of the dermal skeleton in stem‐ and early crown‐osteichthyans.

## Author Contributions

**Xianren Shan:** investigation, data curation, formal analysis, visualization, writing – original draft, writing – review and editing. **Edine Pape:** investigation, data curation, formal analysis, visualization, writing – original draft. **Joseph N. Keating:** methodology, supervision, writing – review and editing. **Martin Rücklin:** resources, supervision, writing – review and editing. **Davide Pisani:** supervision, writing – review and editing. **Philip C. J. Donoghue:** conceptualization, methodology, data curation, investigation, formal analysis, supervision, funding acquisition, project administration, resources, writing – review and editing.

## Conflicts of Interest

The authors declare no conflicts of interest.

## Supporting information

S1.

S2.

## Data Availability

Data are available at the University of Bristol data repository, data.bris, at https://doi.org/10.5523/bris.382c6rmj259rr1zryyuk9rffl0.
