# Supplementary material for: The Dermal Skeleton of Stem‐Actinopterygian Moythomasia durgaringa and Its Implications for the Nature of the Ancestral Osteichthyan
Source: J Morphol. 2026 Mar 19;287(3):e70120. doi: 10.1002/jmor.70120 (PMC13003200; doi:10.1002/jmor.70120)
Supplement: Supplementary file 1 — S1. [file JMOR-287-e70120-s001.docx]

**Supplementary Data. Character data and stratigraphic data used in the ancestral state estimation, and results of the analyses.**

**Supplementary Data: Table 1.**

**Character matrix of cranial dermal skeleton and references used for ancestral state estimation**

| Taxon for tree 1 | cranial dermal skeleton | enamel | enamel layer | vascular canal | odontogenic resorption | Reference |
| --- | --- | --- | --- | --- | --- | --- |
| Galeaspida | 1 | 0 | – | – | 0 | Wang et al., 2005 |
| Osteostraci | 1 | 0 | – | 2 | 0 | O’Shea et al.,2019 |
| *Acanthodes* | 1 | 0 | – | 0 | 0 | – |
| *Akmonistion* | 1 | 0 | – | 0 | 0 | – |
| *Austroptyctodus* | 1 | 0 | – | ? | 0 |  |
| *Bothriolepis* | 1 | 0 | – | – | 0 | Downs and Donoghue, 2009 |
| *Brachyacanthus* | 1 | 0 | – | ? | 0 | – |
| *Brindabellaspis* | 1 | 0 | – | ? | 0 | – |
| *Brochoadmones* | 1 | 0 | – | ? | 0 | Hanke and Wilson, 2006 |
| *Buchanosteus* | 1 | 0 | – | ? | 0 | Burrow and Turner, 1999 |
| *Campbellodus* | 1 | 0 | – | ? | 0 | – |
| *Cassidiceps* | 1 | 0 | – | ? | 0 | – |
| *Cheiracanthus* | 1 | 0 | – | 0 | 0 | Burrow et al., 2020 |
| *Cheirolepis* | 1 | 1 | 1 | 1 | 1 | Lu et al., 2016 |
| *Chondrenchelys* | 1 | 0 | – | 0 | 0 | – |
| *Cladodoides* | 1 | 0 | – | 0 | 0 | – |
| *Cladoselache* | 1 | 0 | – | 0 | 0 | – |
| *Climatius* | 1 | 0 | – | 0 | 0 | Burrow et al., 2015 |
| *Cobelodus* | 1 | 0 | – | 0 | 0 | – |
| *Coccosteus* | 1 | 0 | – | ? | 0 | – |
| *Cowralepis* | 1 | 0 | – | ? | 0 | – |
| *Culmacanthus* | 1 | 0 | – | 0 | 0 | – |
| *Debeerius* | 1 | 0 | – | 0 | 0 | – |
| *Dialipina* | 1 | ? | ? | ? | ? | – |
| *Dicksonosteus* | 1 | 0 | – | ? | 0 | – |
| *Diplacanthus* | 1 | 0 | – | 0 | 0 | Burrow et al., 2016 |
| *Doliodus* | 1 | 0 | – | 0 | 0 | – |
| *Entelognathus* | 1 | 0 | – | 0 | 0 | – |
| *Eusthenopteron* | 1 | ? | ? | ? | ? | Zylberberg et al., 2010 |
| *Euthacanthus* | 1 | 0 | – | 0 | 0 | Newman et al., 2014 |
| *Gladiobranchus* | 1 | 0 | – | 0 | 0 | Hanke and Davis, 2008 |
| *Gogonasus* | 1 | ? | ? | ? | ? | Long et al., 1997 |
| *Guiyu* | 1 | 1 | 1 | ? | ? | Zhu et al., 2009 |
| *Hamiltonichthys* | 1 | 0 | – | 0 | 0 | – |
| *Homalacanthus* | 1 | 0 | – | 0 | 0 | Burrow et al., 2022 |
| *Howqualepis* | 1 | ? | ? | ? | ? | – |
| *Incisoscutum* | 1 | 0 | – | – | 0 | Giles et al., 2013 |
| *Ischnacanthus* | 1 | 0 | – | 0 | 0 | Burrow et al., 2018 |
| *Kathemacanthus* | 1 | 0 | – | 0 | 0 | – |
| *Ligulalepis* | 1 | 1 | 1 | 1 | 1 | Burrow et al., 2023 |
| *Lunaspis* | 1 | 0 | – | – | 0 | Giles et al., 2013 |
| *Lupopsyrus* | 1 | 0 | – | 0 | 0 | Hanke and Davis, 2012 |
| *Macropetalichthys* | 1 | 0 | – | 0 | 0 | Burrow and Turner, 1999 |
| *Mesacanthus* | 1 | 0 | – | 0 | 0 | – |
| *Mimipiscis* | 1 | ? | ? | ? | ? | – |
| *Moythomasia* | 1 | 1 | 1 | 1 | 1 | this study |
| *Obtusacanthus* | 1 | 0 | – | 0 | 0 | – |
| *Onychodus* | 1 | 1 | 1 | 1 | ? | Andrews et al., 2005 |
| *Onychoselache* | 1 | 0 | – | 0 | 0 | – |
| *Orthacanthus* | 1 | 0 | – | 0 | 0 | Soler-Gijón, 1999; Beck et al., 2016 |
| *Parexus* | 1 | 0 | – | 0 | 0 | Burrow et al., 2013 |
| *Poracanthodes* | 1 | 0 | – | 0 | 0 | Valiukevicius, 1992 |
| *Porolepis* | 1 | 1 | 0 | 2 | 1 | Gross, 1956; Chang and Smith, 1992 |
| *Promesacanthus* | 1 | 0 | – | 0 | 0 | Hanke, 2008 |
| *Psarolepis* | 1 | 1 | 1 | 1 | 1 | Qu et al., 2013; 2017 |
| *Pterichthyodes* | 1 | 0 | – | ? | 0 | – |
| *Ptomacanthus* | 0 | – | – | – | – | Brazeau, 2012 |
| *Pucapampella* | 1 | 0 | – | 0 | 0 | – |
| *Rhamphodopsis* | 1 | 0 | – | ? | 0 | – |
| *Romundina* | 1 | 0 | – | 0 | 0 | Giles et al., 2013 |
| *Styloichthys* | 1 | 1 | 0 | 2 | 1 | Zhu et al., 2006; 2010; Cui et al., 2025 |
| *Tamiobatis* | 1 | 0 | – | 0 | 0 | – |
| *Tetanopsyrus* | 1 | 0 | – | 0 | 0 | Gagnier et al., 1999 |
| *Tristychius* | 1 | 0 | – | 0 | 0 | – |
| *Parayunnanolepis* | 1 | 0 | – | ? | 0 | – |
| *Sinolepis* | 1 | 0 | – | ? | 0 | – |
| *Quasipetalichthys* | 1 | 0 | – | ? | 0 | – |
| *Diandongpetal–ichthys* | 1 | 0 | – | ? | 0 | – |
| *Gavinaspis* | 1 | 0 | – | ? | 0 | – |
| *Sigaspis* | 1 | 0 | – | ? | 0 | – |
| *Eurycaraspis* | 1 | 0 | – | ? | 0 | – |
| *Gemuendina* | 1 | 0 | – | ? | 0 | – |
| *Glyptolepis* | 1 | ? | ? | ? | ? | Ørvig, 1957 |
| *Gyracanthides* | 1 | 0 | – | 0 | 0 | Turner et al., 2005 |
| *Helodus* | 1 | 0 | – | 0 | 0 | – |
| *Jagorina* | 1 | 0 | – | ? | 0 | – |
| *Janusiscus* | 1 | 0 | – | 0 | 0 | – |
| *Kentuckia* | 1 | ? | ? | ? | ? | – |
| *Kujdanowiaspis* | 1 | 0 | – | ? | 0 | – |
| *Latviacanthus* | 1 | 0 | – | 0 | 0 | Schultze et al., 1982 |
| *Ramirosuarezia* | 1 | 0 | – | 0 | 0 | – |
| *Yunnanolepis* | 1 | 0 | – | 0 | 0 | Giles et al., 2013 |
| *Vernicomacanthus* | 1 | 0 | – | 0 | 0 | Dearden et al., 2021 |
| *Youngolepis* | 1 | 1 | 0 | 2 | ? | Chang and Smith, 1992 |
| *Microbrachius* | 1 | 0 | – | ? | 0 | – |
| *Remigolepis* | 1 | 0 | – | ? | 0 | – |
| *Materpiscis* | 1 | 0 | – | ? | 0 | – |
| *Wuttagoonaspis* | 1 | 0 | – | ? | 0 | – |
| *Groenlandaspis* | 1 | 0 | – | ? | 0 | – |
| *Holonema* | 1 | 0 | – | - | 0 | Giles et al., 2013 |
| *Parabuchanosteus* | 1 | 0 | – | ? | 0 | Burrow and Turner, 1999 |
| *Compagopiscis* | 1 | 0 | – | - | 0 | Giles et al., 2013 |
| *Eastmanosteus* | 1 | 0 | – | ? | 0 | – |
| *Achoania* | 1 | 1 | ? | ? | ? | Zhu et al., 2001 |
| *Rhadinacanthus* | 1 | 0 | – | 0 | 0 | Burrow et al., 2016 |
| *Diabolepis* | 1 | 1 | 0 | 2 | ? | Chang and Smith, 1992 |
| *Osteolepis* | 1 | ? | ? | ? | ? | Gross, 1956; Schultze, 2016 |
| *Osorioichthys* | 1 | ? | ? | ? | ? | – |
| *Kenichthys* | 1 | 1 | ? | ? | ? | Chang and Zhu, 1993 |
| *Lophosteus* | 1 | 0 | – | 0 | 0 | Gross, 1969 |
| *Meemannia* | 1 | 1 | 1 | 1 | ? | Zhu et al., 2010; Lu et al., 2016 |
| *Miguashaia* | 1 | ? | ? | ? | ? | Fernández et al., 2021 |
| *Powichthys* | 1 | 1 | 0 | 2 | ? | Chang and Smith, 1992 |

**Supplementary Data: Table 2.**

**Character matrix of postcranial dermal skeleton and references used for ancestral state estimation**

| Taxon for tree 1 | cranial dermal skeleton | enamel | enamel layer | vascular canal | odontogenic resorption | Reference |
| --- | --- | --- | --- | --- | --- | --- |
| Galeaspida | 1 | 0 | – | – | 0 | Wang et al., 2005 |
| Osteostraci | 1 | 0 | – | 2 | 0 | O’Shea et al., 2019 |
| *Acanthodes* | 1 | 0 | – | 0 | 0 | Gross, 1935 |
| *Akmonistion* | 1 | 0 | – | 0 | 0 | – |
| *Austroptyctodus* | 1 | 0 | – | 0 | 0 | – |
| *Bothriolepis* | 0 | – | – | – | – | – |
| *Brachyacanthus* | 1 | 0 | – | 0 | 0 | – |
| *Brindabellaspis* | 1 | 0 | – | 0 | 0 | – |
| *Brochoadmones* | 1 | 0 | – | 0 | 0 | Hanke and Wilson, 2006 |
| *Buchanosteus* | 1 | 0 | – | 0 | 0 | Burrow and Turner, 1998 |
| *Campbellodus* | 1 | 0 | – | 0 | 0 | – |
| *Cassidiceps* | 1 | 0 | – | 0 | 0 | – |
| *Cheiracanthus* | 1 | 0 | – | 0 | 0 | Denison, 1979; Burrow et al., 2020 |
| *Cheirolepis* | 1 | 1 | 1 | 0 | 1 | Zylberberg et al., 2015 |
| *Chondrenchelys* | 1 | 0 | – | 0 | 0 | – |
| *Cladodoides* | ? | ? | ? | ? | ? | – |
| *Cladoselache* | ? | ? | ? | ? | ? | – |
| *Climatius* | 1 | 0 | – | 0 | 0 | Burrow et al., 2015 |
| *Cobelodus* | ? | ? | ? | ? | ? | – |
| *Coccosteus* | 1 | 0 | – | ? | 0 | – |
| *Cowralepis* | 0 | – | – | – | – | – |
| *Culmacanthus* | 1 | 0 | – | 0 | 0 | – |
| *Debeerius* | 1 | 0 | – | 0 | 0 | – |
| *Dialipina* | 1 | 1 | 1 | 0 | ? | Schultze, 1968 |
| *Dicksonosteus* | ? | ? | ? | ? | ? | – |
| *Diplacanthus* | 1 | 0 | – | 0 | 0 | Denison, 1979; Burrow et al., 2016 |
| *Doliodus* | 1 | 0 | – | 0 | 0 | – |
| *Entelognathus* | 1 | 0 | – | 0 | 0 | Cui et al., 2025 |
| *Eusthenopteron* | 1 | 0 | – | ? | 0 | Zylberberg et al., 2010 |
| *Euthacanthus* | 1 | 0 | – | 0 | 0 | Denison, 1979; Newman et al., 2014 |
| *Gladiobranchus* | 1 | 0 | – | 0 | 0 | Hanke and Davis, 2008 |
| *Gogonasus* | 1 | 1 | 0 | 2 | ? | Long et al., 1997 |
| *Guiyu* | 1 | 1 | 1 | ? | ? | – |
| *Hamiltonichthys* | 1 | 0 | – | 0 | 0 | – |
| *Homalacanthus* | 1 | 0 | – | 0 | 0 | Denison, 1979; Burrow et al., 2022 |
| *Howqualepis* | 1 | 1 | 1 | ? | ? | – |
| *Incisoscutum* | 0 | – | – | – | – | – |
| *Ischnacanthus* | 1 | 0 | – | ? | 0 | Burrow et al., 2018 |
| *Kathemacanthus* | 1 | 0 | – | 0 | 0 | – |
| *Ligulalepis* | 1 | 1 | 1 | 1 | 1 | Schultze, 1968; Burrow et al., 2023 |
| *Lunaspis* | 1 | 0 | – | 0 | 0 | Giles et al., 2013 |
| *Lupopsyrus* | 1 | 0 | – | 0 | 0 | Hanke and Davis, 2012 |
| *Macropetalichthys* | ? | ? | ? | ? | ? | Burrow and Turner, 1999 |
| *Mesacanthus* | 1 | 0 | – | 0 | 0 | – |
| *Mimipiscis* | 1 | 1 | 1 | 0 | 1 | Gagnier, 1984 |
| *Moythomasia* | 1 | 1 | 1 | 0 | 1 | this study |
| *Obtusacanthus* | 1 | 0 | – | 0 | 0 | – |
| *Onychodus* | 1 | 1 | 1 | 1 | 1 | – |
| *Onychoselache* | 1 | 0 | – | 0 | 0 | – |
| *Orthacanthus* | 1 | 0 | – | 0 | 0 | Soler-Gijón, 1999; Beck et al., 2016 |
| *Parexus* | 1 | 0 | – | 0 | 0 | Burrow et al., 2013 |
| *Poracanthodes* | 1 | 0 | – | 2 | 0 | Valiukevicius, 1992 |
| *Porolepis* | 1 | 1 | 1 | 2 | 1 | Gross, 1956; Chang and Smith,1992 |
| *Promesacanthus* | 1 | 0 | – | 0 | 0 | Hanke, 2008 |
| *Psarolepis* | 1 | 1 | 1 | 1 | 1 | Qu et al., 2013; 2017 |
| *Pterichthyodes* | 1 | 0 | – | ? | 0 | – |
| *Ptomacanthus* | 1 | 0 | – | 0 | 0 | Brazeau, 2012 |
| *Pucapampella* | ? | ? | ? | ? | ? | – |
| *Rhamphodopsis* | ? | ? | ? | ? | ? | – |
| *Romundina* | 1 | 0 | – | 0 | 0 | Giles et al., 2013; Burrow and Turner, 1999 |
| *Styloichthys* | 1 | 1 | 0 | 2 | 1 | Zhu et al., 2006; 2010; Cui et al., 2025 |
| *Tamiobatis* | 1 | 0 | – | 0 | 0 | – |
| *Tetanopsyrus* | 1 | 0 | – | 0 | 0 | Gagnier et al., 1999 |
| *Tristychius* | 0 | – | – | – | – | – |
| *Parayunnanolepis* | 1 | 0 | – | ? | 0 | – |
| *Sinolepis* | 1 | 0 | – | ? | 0 | – |
| *Quasipetalichthys* | ? | ? | ? | ? | ? | – |
| *Diandongpetal–ichthys* | ? | ? | ? | ? | ? | – |
| *Gavinaspis* | ? | ? | ? | ? | ? | – |
| *Sigaspis* | 1 | 0 | – | ? | 0 | – |
| *Eurycaraspis* | ? | ? | ? | ? | ? | – |
| *Gemuendina* | 1 | 0 | – | ? | 0 | – |
| *Glyptolepis* | 1 | ? | ? | ? | ? | – |
| *Gyracanthides* | 1 | 0 | – | 0 | 0 | Turner et al., 2005 |
| *Helodus* | 1 | 0 | – | 0 | 0 | – |
| *Jagorina* | 1 | 0 | – | ? | 0 | – |
| *Janusiscus* | ? | ? | ? | ? | ? | – |
| *Kentuckia* | ? | ? | ? | ? | ? | – |
| *Kujdanowiaspis* | ? | ? | ? | ? | ? | – |
| *Latviacanthus* | 1 | 0 | – | 0 | 0 | Schultze et al., 1982 |
| *Ramirosuarezia* | ? | ? | ? | ? | ? | – |
| *Yunnanolepis* | 1 | 0 | – | ? | 0 | – |
| *Vernicomacanthus* | 1 | 0 | – | 0 | 0 | Dearden et al., 2021 |
| *Youngolepis* | ? | ? | ? | ? | ? | – |
| *Microbrachius* | ? | ? | ? | ? | ? | – |
| *Remigolepis* | 1 | 0 | – | ? | 0 | – |
| *Materpiscis* | ? | ? | ? | ? | ? | – |
| *Wuttagoonaspis* | 1 | 0 | – | ? | 0 | – |
| *Groenlandaspis* | ? | ? | ? | ? | ? | – |
| *Holonema* | 1 | 0 | – | 0 | 0 | Giles et al., 2013 |
| *Parabuchanosteus* | 1 | 0 | – | 0 | 0 | Burrow and Turner, 1998 |
| *Compagopiscis* | 1 | 0 | – | ? | 0 | Giles et al., 2013 |
| *Eastmanosteus* | ? | ? | ? | ? | ? | – |
| *Achoania* | ? | ? | ? | ? | ? | – |
| *Rhadinacanthus* | ? | ? | ? | ? | ? | Burrow et al., 2016 |
| *Diabolepis* | ? | ? | ? | ? | ? | – |
| *Osteolepis* | 1 | 1 | 0 | 2 | ? | Gross, 1956, Schultze, 2016 |
| *Osorioichthys* | ? | ? | ? | ? | ? | – |
| *Kenichthys* | ? | ? | ? | ? | ? | – |
| *Lophosteus* | 1 | 0 | – | 0 | 0 | Jerve et al., 2016 |
| *Meemannia* | 1 | ? | ? | ? | ? | – |
| *Miguashaia* | 1 | 1 | 1 | 1 | 1 | Fernández et al., 2021 |
| *Powichthys* | ? | ? | ? | ? | ? | – |

**Supplementary Data: Table 3.**

**Character matrix of cranial dermal skeleton and references used for ancestral state estimation**

| Taxon for trees 2 and 3 | cranial dermal skeleton | enamel | enamel layer | vascular canal | odontogenic resorption | Reference |
| --- | --- | --- | --- | --- | --- | --- |
| *Hemicyclaspis murchisoni* | 1 | 0 | – | 2 | 0 | – |
| *Cephalaspis lyelli* | 1 | 0 | – | 2 | 0 | – |
| *Zenaspis salweyi* | 1 | 0 | – | 2 | 0 | – |
| *Benneviaspis holtedahli* | 1 | 0 | – | ? | 0 | – |
| *Boreaspis macrorhynchus* | 1 | 0 | – | ? | 0 | – |
| *Norselaspis glacialis* | 1 | 0 | – | ? | 0 | – |
| *Nectaspis areolate* | 1 | 0 | – | ? | 0 | – |
| *Procephalaspis oeselensis* | 1 | 0 | – | 2 | 0 | Bremer et al., 2021 |
| *Tremataspis mammillata* | 1 | 0 | – | 2 | 0 | O’Shea et al., 2019 |
| *Waengsjoeaspis excellens* | 1 | 0 | – | 2 | 0 | – |
| *Escuminaspis laticeps* | 1 | 0 | – | 1 | 0 | – |
| *Eugaleaspis changi* | 1 | 0 | – | – | 0 | – |
| *Hanyangaspis guodingshanensis* | 1 | 0 | – | – | 0 | Wang et al., 2005 |
| *Polybranchiaspis liaojiaoshanensis* | 1 | 0 | – | – | 0 | – |
| *Bannhuanaspis vukhuci* | 1 | 0 | – | – | 0 | – |
| *Wenshanaspis zhichangensis* | 1 | 0 | – | – | 0 | – |
| *Shuyu zhejiangensis* | 1 | 0 | – | – | 0 | – |
| *Polybranchiaspid sp histological samples* | 1 | 0 | – | – | 0 | Wang et al., 2005 |
| *Yunnanolepis sp* | 1 | 0 | – | 0 | 0 | Giles et al., 2013 |
| *Parayunnanolepis xitunensis* | 1 | 0 | – | ? | 0 | – |
| *Microbrachius dicki* | 1 | 0 | – | ? | 0 | – |
| *Bothriolepis sp Gogo* | 1 | 0 | – | – | 0 | Downs and Donoghue, 2009 |
| *Bothriolepis canadensis* | 1 | 0 | – | – | 0 | Downs and Donoghue, 2009 |
| *Pterichthyodes milleri* | 1 | 0 | – | ? | 0 | – |
| *Remigolepis walkeri* | 1 | 0 | – | ? | 0 | – |
| *Diandongpetalichthys liaojiaoshanensis* | 1 | 0 | – | ? | 0 | – |
| *Quasipetalichthys haikouensis* | 1 | 0 | – | ? | 0 | – |
| *Eurycaraspis incilis* | 1 | 0 | – | ? | 0 | – |
| *Lunaspis broili* | 1 | 0 | – | – | 0 | Giles et al., 2013 |
| *Macropetalichthys rapheidolabis* | 1 | 0 | – | 0 | 0 | Burrow and Turner, 1999 |
| *Wuttagoonaspis fletcheri* | ? | 0 | – | ? | 0 | – |
| *Cowralepis mclachlani* | 1 | 0 | – | ? | 0 | – |
| *Gavinaspis convergens* | 1 | 0 | – | ? | 0 | – |
| *Sigaspis lepidophora* | 1 | 0 | – | ? | 0 | – |
| *Kujdanowiaspis podolica* | 1 | 0 | – | ? | 0 | – |
| *Dicksonosteus arcticus* | 1 | 0 | – | ? | 0 | – |
| *Groenlandaspis sp Mt Howitt* | 1 | 0 | – | ? | 0 | – |
| *Buchanosteus confertituberculatus* | 1 | 0 | – | ? | 0 | Burrow and Turner, 1999 |
| *Parabuchanosteus murrumbidgeensis* | 1 | 0 | – | ? | 0 | – |
| *Holonema westolli* | 1 | 0 | – | – | 0 | Giles et al., 2013 |
| *Coccosteus cuspidatus* | 1 | 0 | – | ? | 0 | – |
| *Incisoscutum ritchiei* | 1 | 0 | – | – | 0 | Giles et al., 2013 |
| *Eastmanosteus calliaspis* | 1 | 0 | – | ? | 0 | – |
| *Compagopiscis croucheri* | 1 | 0 | – | – | 0 | Giles et al., 2013 |
| *Materpiscis attenboroughi* | 1 | 0 | – | ? | 0 | – |
| *Austroptyctodus gardineri* | 1 | 0 | – | ? | 0 | – |
| *Campbellodus decipiens* | 1 | 0 | – | ? | 0 | – |
| *Rhamphodopsis threiplandi* | 1 | 0 | – | ? | 0 | – |
| *Brindabellaspis stensioi* | 1 | 0 | – | ? | 0 | – |
| *Romundina stellina* | 1 | 0 | – | 0 | 0 | Giles et al., 2013 |
| *Jagorina pandora* | 1 | 0 | – | 0 | 0 | – |
| *Gemuendina stuertzi* | 1 | 0 | – | 0 | 0 | – |
| *Entelognathus primordialis* | 1 | 0 | – | 0 | 0 | – |
| *Janusiscus schultzei* | 1 | 0 | – | 0 | 0 | – |
| *Ramirosuarezia boliviana* | 1 | 0 | – | 0 | 0 | – |
| *Acanthodes bronni* | 1 | 0 | – | 0 | 0 | – |
| *Brachyacanthus scutiger* | 1 | 0 | – | 0 | 0 | – |
| *Brochoadmones milesi* | 1 | 0 | – | 0 | 0 | Hanke and Wilson, 2006 |
| *Cassidiceps vermiculatus* | 1 | 0 | – | 0 | 0 | – |
| *Cheiracanthus sp* | 1 | 0 | – | 0 | 0 | Burrow et al., 2020 |
| *Climatius reticulatus* | 1 | 0 | – | 0 | 0 | Burrow et al., 2015 |
| *Culmacanthus stewarti* | 1 | 0 | – | 0 | 0 | – |
| *Euthacanthus macnicoli* | 1 | 0 | – | 0 | 0 | Newman et al., 2014 |
| *Gladiobranchus probaton* | 1 | 0 | – | 0 | 0 | Hanke and Davis, 2008 |
| *Homalacanthus concinnus* | 1 | 0 | – | 0 | 0 | Burrow et al., 2022 |
| *Ischnacanthus gracilis* | 1 | 0 | – | 0 | 0 | Burrow et al., 2018 |
| *Kathemacanthus rosulentus* | 1 | 0 | – | 0 | 0 | – |
| *Latviacanthus ventspilsensis* | 1 | 0 | – | 0 | 0 | Schultze et al., 1982 |
| *Lupopsyrus pygmaeus* | 1 | 0 | – | 0 | 0 | Hanke and Davis, 2012 |
| *Mesacanthus mitchelli* | 1 | 0 | – | ? | 0 | – |
| *Obtusacanthus corroconis* | 1 | 0 | – | 0 | 0 | – |
| *Parexus recurvus* | 1 | 0 | – | 0 | 0 | Burrow et al., 2013 |
| *Poracanthodes menneri* | 1 | 0 | – | ? | 0 | Valiukevicius, 1992 |
| *Promesacanthus eppleri* | 1 | 0 | – | 0 | 0 | Hanke, 2008 |
| *Ptomacanthus anglicus* | 1 | 0 | – | 0 | 0 | Brazeau, 2012 |
| *Diplacanthus striatus* | 1 | 0 | – | 0 | 0 | Burrow et al., 2016 |
| *Tetanopsyrus lindoei breviacanthias* | 1 | 0 | – | 0 | 0 | Gagnier et al., 1999 |
| *Vernicomacanthus waynensis* | 1 | 0 | – | 0 | 0 | – |
| *Cladodoides wildungensis* | 1 | 0 | – | 0 | 0 | – |
| *Akmonistion zangerli* | 1 | 0 | – | 0 | 0 | – |
| *Cobelodus braincase* | 1 | 0 | – | ? | 0 | – |
| *Cladoselache kepleri fyleri* | 1 | 0 | – | ? | 0 | – |
| *Chondrenchelys problematica* | 1 | 0 | – | ? | 0 | – |
| *Helodus simplex* | 1 | 0 | – | ? | 0 | – |
| *Debeerius ellefseni* | 1 | 0 | – | – | 0 | – |
| *Doliodus problematicus* | 1 | 0 | – | ? | 0 | – |
| *Hamiltonichthys mapesi* | 1 | 0 | – | ? | 0 | – |
| *Onychoselache traquari* | 1 | 0 | – | ? | 0 | – |
| *Orthacanthus sp* | 1 | 0 | – | – | 0 | Soler-Gijón, 1999; Beck et al., 2016 |
| *Pucapampella rodrigae* | 1 | 0 | – | ? | 0 | – |
| *Tamiobatis vetustus* | 1 | 0 | – | ? | 0 | – |
| *Tristychius arcuatus* | 1 | 0 | – | ? | 0 | – |
| *Dialipina salgueiroensis* | 1 | ? | ? | ? | ? | – |
| *Ligulalepis toombsi* | 1 | 1 | 1 | 1 | 1 | Burrow et al., 2023 |
| *Cheirolepis canadensis* | 1 | 1 | 1 | 1 | 1 | Lu et al., 2016 |
| *Cheirolepis trailli* | 1 | 1 | 1 | 1 | 1 | Lu et al., 2016 |
| *Howqualepis rostridens* | 1 | ? | ? | ? | ? | – |
| *Mimipiscis toombsi* | 1 | ? | ? | ? | ? | – |
| *Moythomasia durgaringa* | 1 | 1 | 1 | 1 | 1 | this study |
| *Kentuckia deani* | 1 | ? | ? | ? | ? | – |
| *Osorioichthys marginis* | 1 | ? | ? | ? | ? | – |
| *Meemannia eos* | 1 | 1 | 1 | 1 | ? | Zhu et al., 2010; Lu et al., 2016 |
| *Guiyu oneiros* | 1 | 1 | 1 | ? | ? | Zhu et al., 2009 |
| *Psarolepis romeri* | 1 | 1 | 1 | 1 | 1 | Qu et al., 2013; 2017 |
| *Achoania jarvikii* | 1 | 1 | ? | ? | ? | – |
| *Onychodus jandemarrai* | 1 | 1 | 1 | 1 | ? | Andrews et al., 2005 |
| *Miguashaia bureaui* | 1 | ? | ? | ? | ? | Fernández et al., 2021 |
| *Styloichthys changae* | 1 | 1 | 0 | 2 | 1 | Zhu et al., 2006; 2010; Cui et al., 2025 |
| *Diabolepis speratus* | 1 | 1 | 0 | 2 | ? | Chang and Smith, 1992 |
| *Youngolepis praecursor* | 1 | 1 | 0 | 2 | ? | Chang and Smith, 1992 |
| *Powichthys thorsteinssoni* | 1 | 1 | 0 | 2 | ? | Chang and Smith, 1992 |
| *Porolepis sp* | 1 | 1 | 0 | 2 | 1 | Gross, 1956; Chang and Smith, 1992 |
| *Glyptolepis groenlandica* | 1 | ? | ? | ? | ? | Ørvig, 1957 |
| *Kenichthys campbelli* | 1 | 1 | ? | ? | ? | Chang and Zhu, 1993 |
| *Osteolepis macrolepidotus* | 1 | ? | ? | ? | ? | Gross, 1956; Schultze, 2016 |
| *Gogonasus andrewsae* | 1 | ? | ? | ? | ? | Long et al., 1997 |
| *Eusthenopteron foordi* | 1 | ? | ? | ? | ? | Zylberberg et al., 2010 |

**Supplementary Data: Table 4.**

**Character matrix of postcranial dermal skeleton and references used for ancestral state estimation**

| Taxon for trees 2 and 3 | cranial dermal skeleton | enamel | enamel layer | canal system | odontogenic resorption | Reference |
| --- | --- | --- | --- | --- | --- | --- |
| *Hemicyclaspis murchisoni* | 1 | 0 | – | 2 | 0 | – |
| *Cephalaspis lyelli* | 1 | 0 | – | 2 | 0 | – |
| *Zenaspis salweyi* | 1 | 0 | – | 2 | 0 | – |
| *Benneviaspis holtedahli* | 1 | 0 | – | ? | 0 | – |
| *Boreaspis macrorhynchus* | 1 | 0 | – | ? | 0 | – |
| *Norselaspis glacialis* | 1 | 0 | – | ? | 0 | – |
| *Nectaspis areolate* | 1 | 0 | – | ? | 0 | – |
| *Procephalaspis oeselensis* | 1 | 0 | – | 2 | 0 | Bremer et al., 2021 |
| *Tremataspis mammillata* | 1 | 0 | – | 2 | 0 | O’Shea et al., 2019 |
| *Waengsjoeaspis excellens* | 1 | 0 | – | 2 | 0 | – |
| *Escuminaspis laticeps* | 1 | 0 | – | 1 | 0 | – |
| *Eugaleaspis changi* | 1 | 0 | – | – | 0 | – |
| *Hanyangaspis guodingshanensis* | 1 | 0 | – | – | 0 | – |
| *Polybranchiaspis liaojiaoshanensis* | 1 | 0 | – | – | 0 | – |
| *Bannhuanaspis vukhuci* | 1 | 0 | – | – | 0 | – |
| *Wenshanaspis zhichangensis* | 1 | 0 | – | – | 0 | – |
| *Shuyu zhejiangensis* | 1 | 0 | – | – | 0 | – |
| *Polybranchiaspid sp histological samples* | 1 | 0 | – | – | 0 | – |
| *Yunnanolepis sp* | 1 | 0 | – | ? | 0 | – |
| *Parayunnanolepis xitunensis* | 1 | 0 | – | ? | 0 | – |
| *Microbrachius dicki* | ? | ? | ? | ? | ? | – |
| *Bothriolepis sp Gogo* | 0 | – | – | – | – | – |
| *Bothriolepis canadensis* | 0 | – | – | – | – | – |
| *Pterichthyodes milleri* | 1 | 0 | – | ? | 0 | – |
| *Remigolepis walkeri* | 1 | 0 | – | ? | 0 | – |
| *Diandongpetalichthys liaojiaoshanensis* | ? | ? | ? | ? | ? | – |
| *Quasipetalichthys haikouensis* | ? | ? | ? | ? | ? | – |
| *Eurycaraspis incilis* | ? | ? | ? | ? | ? | – |
| *Lunaspis broili* | 1 | 0 | – | 0 | 0 | Giles et al., 2013 |
| *Macropetalichthys rapheidolabis* | ? | ? | ? | ? | ? | Burrow and Turner, 1999 |
| *Wuttagoonaspis fletcheri* | 1 | 0 | – | ? | 0 | – |
| *Cowralepis mclachlani* | 0 | – | – | – | – | – |
| *Gavinaspis convergens* | ? | ? | ? | ? | ? | – |
| *Sigaspis lepidophora* | 1 | 0 | – | ? | 0 | – |
| *Kujdanowiaspis podolica* | ? | ? | ? | ? | ? | – |
| *Dicksonosteus arcticus* | ? | ? | ? | ? | ? | – |
| *Groenlandaspis sp Mt Howitt* | ? | ? | ? | ? | ? | – |
| *Buchanosteus confertituberculatus* | 1 | 0 | – | 0 | 0 | Burrow and Turner, 1998 |
| *Parabuchanosteus murrumbidgeensis* | 1 | 0 | – | 0 | 0 | Burrow and Turner, 1998 |
| *Holonema westolli* | 1 | 0 | – | 0 | 0 | Giles et al., 2013 |
| *Coccosteus cuspidatus* | 1 | 0 | – | ? | 0 | – |
| *Incisoscutum ritchiei* | 0 | – | – | – | – | – |
| *Eastmanosteus calliaspis* | ? | ? | ? | ? | ? | – |
| *Compagopiscis croucheri* | 1 | 0 | – | ? | 0 | Giles et al., 2013 |
| *Materpiscis attenboroughi* | ? | ? | ? | ? | ? | – |
| *Austroptyctodus gardineri* | 1 | 0 | – | 0 | 0 | – |
| *Campbellodus decipiens* | 1 | 0 | – | 0 | 0 | – |
| *Rhamphodopsis threiplandi* | ? | ? | ? | ? | ? | – |
| *Brindabellaspis stensioi* | 1 | 0 | – | 0 | 0 | – |
| *Romundina stellina* | 1 | 0 | – | 0 | 0 | Giles et al., 2013; Burrow and Turner, 1999 |
| *Jagorina pandora* | 1 | 0 | – | ? | 0 | – |
| *Gemuendina stuertzi* | 1 | 0 | – | ? | 0 | – |
| *Entelognathus primordialis* | 1 | 0 | – | 0 | 0 | Cui et al., 2025 |
| *Janusiscus schultzei* | ? | ? | ? | ? | ? | – |
| *Ramirosuarezia boliviana* | ? | ? | ? | ? | ? | – |
| *Acanthodes bronni* | 1 | 0 | – | 0 | 0 | Gross, 1935 |
| *Brachyacanthus scutiger* | 1 | 0 | – | 0 | 0 | – |
| *Brochoadmones milesi* | 1 | 0 | – | 0 | 0 | Hanke and Wilson, 2006 |
| *Cassidiceps vermiculatus* | 1 | 0 | – | 0 | 0 | – |
| *Cheiracanthus sp* | 1 | 0 | – | 0 | 0 | Denison, 1979; Burrow et al., 2020 |
| *Climatius reticulatus* | 1 | 0 | – | 0 | 0 |  |
| *Culmacanthus stewarti* | 1 | 0 | – | 0 | 0 | Burrow et al., 2015 |
| *Euthacanthus macnicoli* | 1 | 0 | – | 0 | 0 | Denison, 1979; Newman et al., 2014 |
| *Gladiobranchus probaton* | 1 | 0 | – | 0 | 0 | Hanke and Davis, 2008 |
| *Homalacanthus concinnus* | 1 | 0 | – | 0 | 0 | Denison, 1979; Burrow et al., 2022 |
| *Ischnacanthus gracilis* | 1 | 0 | – | 0 | 0 | Burrow et al., 2018 |
| *Kathemacanthus rosulentus* | 1 | 0 | – | 0 | 0 | – |
| *Latviacanthus ventspilsensis* | 1 | 0 | – | 0 | 0 | Schultze et al., 1982 |
| *Lupopsyrus pygmaeus* | 1 | 0 | – | 0 | 0 | Hanke and Davis, 2012 |
| *Mesacanthus mitchelli* | 1 | 0 | – | 0 | 0 | – |
| *Obtusacanthus corroconis* | 1 | 0 | – | 0 | 0 | – |
| *Parexus recurvus* | 1 | 0 | – | 0 | 0 | Burrow et al., 2013 |
| *Poracanthodes menneri* | 1 | 0 | – | 2 | 0 | Valiukevicius, 1992 |
| *Promesacanthus eppleri* | 1 | 0 | – | 0 | 0 | Hanke, 2008 |
| *Ptomacanthus anglicus* | 1 | 0 | – | 0 | 0 | Brazeau, 2012 |
| *Diplacanthus striatus* | 1 | 0 | – | 0 | 0 | Denison, 1979; Burrow et al., 2016 |
| *Tetanopsyrus lindoei breviacanthias* | 1 | 0 | – | 0 | 0 | Gagnier et al., 1999 |
| *Vernicomacanthus waynensis* | 1 | 0 | – | 0 | 0 | Dearden et al., 2021 |
| *Cladodoides wildungensis* | ? | ? | ? | ? | ? | – |
| *Akmonistion zangerli* | 1 | 0 | – | 0 | 0 | – |
| *Cobelodus braincase* | ? | ? | ? | ? | ? | – |
| *Cladoselache kepleri fyleri* | ? | ? | ? | ? | ? | – |
| *Chondrenchelys problematica* | 1 | 0 | – | 0 | 0 | – |
| *Helodus simplex* | 1 | 0 | – | 0 | 0 | – |
| *Debeerius ellefseni* | 1 | 0 | – | 0 | 0 | – |
| *Doliodus problematicus* | 1 | 0 | – | 0 | 0 | – |
| *Hamiltonichthys mapesi* | 1 | 0 | – | 0 | 0 | – |
| *Onychoselache traquari* | 1 | 0 | – | 0 | 0 | – |
| *Orthacanthus sp* | 1 | 0 | – | 0 | 0 | Soler-Gijón, 1999; Beck et al., 2016 |
| *Pucapampella rodrigae* | ? | ? | ? | ? | ? | – |
| *Tamiobatis vetustus* | 1 | 0 | – | 0 | 0 | – |
| *Tristychius arcuatus* | 0 | – | – | – | – | – |
| *Dialipina salgueiroensis* | 1 | 1 | 1 | 0 | ? | Schultze, 1968 |
| *Ligulalepis toombsi* | 1 | 1 | 1 | 1 | 1 | Schultze, 1968; Burrow et al., 2023 |
| *Cheirolepis canadensis* | 1 | 1 | 1 | 0 | 1 | Zylberberg et al., 2015 |
| *Cheirolepis trailli* | 1 | 1 | 1 | 0 | 1 | Zylberberg et al., 2015 |
| *Howqualepis rostridens* | 1 | 1 | 1 | ? | ? | – |
| *Mimipiscis toombsi* | 1 | 1 | 1 | 0 | 1 | Gagnier, 1984 |
| *Moythomasia durgaringa* | 1 | 1 | 1 | 0 | 1 | this study |
| *Kentuckia deani* | ? | ? | ? | ? | ? | – |
| *Osorioichthys marginis* | ? | ? | ? | ? | ? | – |
| *Meemannia eos* | 1 | ? | ? | ? | ? | – |
| *Guiyu oneiros* | 1 | 1 | 1 | ? | ? | – |
| *Psarolepis romeri* | 1 | 1 | 1 | 1 | 1 | Qu et al., 2013; 2017 |
| *Achoania jarvikii* | ? | ? | ? | ? | ? | – |
| *Onychodus jandemarrai* | 1 | 1 | 1 | 1 | 1 | – |
| *Miguashaia bureaui* | 1 | 1 | 1 | 1 | 1 | Fernández et al., 2021 |
| *Styloichthys changae* | 1 | 1 | 0 | 2 | 1 | Zhu et al., 2006; 2010; Cui et al., 2025 |
| *Diabolepis speratus* | ? | ? | ? | ? | ? | – |
| *Youngolepis praecursor* | ? | ? | ? | ? | ? | – |
| *Powichthys thorsteinssoni* | ? | ? | ? | ? | ? | – |
| *Porolepis sp* | 1 | 1 | 1 | 2 | 1 | Gross, 1956; Chang and Smith,1992 |
| *Glyptolepis groenlandica* | 1 | ? | ? | ? | ? | – |
| *Kenichthys campbelli* | ? | ? | ? | ? | ? | – |
| *Osteolepis macrolepidotus* | 1 | 1 | 0 | 2 | ? | Gross, 1956, Schultze, 2016 |
| *Gogonasus andrewsae* | 1 | 1 | 0 | 2 | ? | Long et al., 1997 |
| *Eusthenopteron foordi* | 1 | 0 | – | ? | 0 | Zylberberg et al., 2010 |

**

**S1**

**S2**

**S3**

**S4**

**S5**

**S6**

**S7**

**S8**

**S9**

**S10**

**S11**

**S12**

**S13**

**S14**

**S15**

**S16**

**S17**

**S18**

**S19**

**S20**

**S21**

**S22**

**S23**

**S24**

**References**

Andrews, M., Long, J., Ahlberg, P., Barwick, R. and Campbell, K., 2005. The structure of the sarcopterygian *Onychodus jandemarrai* n. sp. from Gogo, Western Australia: with a functional interpretation of the skeleton. Earth and Environmental Science Transactions of the Royal Society of Edinburgh, 96(3): 197–307.

Beck, K.G., Soler-Gijón, R., Carlucci, J.R. and Willis, R.E., 2016. Morphology and histology of dorsal spines of the xenacanthid shark *Orthacanthus platypternus* from the Lower Permian of Texas, USA: Palaeobiological and palaeoenvironmental implications. Acta Palaeontologica Polonica, 61(1): 97–117.

Brazeau, M.D., 2012. A revision of the anatomy of the Early Devonian jawed vertebrate *Ptomacanthus anglicus* Miles. Palaeontology, 55(2): 355–367.

Bremer, O., Qu, Q., Sanchez, S., Märss, T., Fernandez, V. and Blom, H., 2021. The emergence of a complex pore‐canal system in the dermal skeleton of *Tremataspis* (Osteostraci). Journal of morphology, 282(8): 1141–1157.

Burrow, C., den Blaauwen, J. and Newman, M., 2020. A redescription of the three longest-known species of the acanthodian *Cheiracanthus* from the Middle Devonian of Scotland.

Burrow, C., den Blaauwen, J., Newman, M., Lodge, V. and Davidson, R., 2016. The diplacanthid fishes (Acanthodii, Diplacanthiformes, Diplacanthidae) from the Middle Devonian of Scotland.

Burrow, C.J., Davidson, R.G., Den Blaauwen, J.L. and Newman, M.J., 2015. Revision of *Climatius reticulatus* Agassiz, 1844 (Acanthodii, Climatiidae), from the Lower Devonian of Scotland, based on new histological and morphological data. Journal of Vertebrate Paleontology, 35(3): e913421.

Burrow, C.J., den Blaauwen, J.L. and Newman, M.J., 2022. New information on the Early Devonian acanthodian *Mesacanthus mitchelli* from the Midland Valley of Scotland. Scottish Journal of Geology, 58(2): sjg2021–004.

Burrow, C.J., Newman, M., Den Blaauwen, J., Jones, R. and Davidson, R., 2018. The early Devonian ischnacanthiform acanthodian *Ischnacanthus gracilis* (Egerton, 1861) from the Midland Valley of Scotland. Acta Geologica Polonica, 68(3): 335–362.

Burrow, C.J., Newman, M.J., Davidson, R.G. and den Blaauwen, J.L., 2013. Redescription of *Parexus recurvus*, an Early Devonian acanthodian from the Midland Valley of Scotland. Alcheringa: an Australasian Journal of Palaeontology, 37(3): 392–414.

Burrow, C.J. and Turner, S., 1999. A review of placoderm scales, and their significance in placoderm phylogeny. Journal of Vertebrate Paleontology, 19(2): 204–219.

Burrow, C.J., Young, G.C. and Lu, J., 2023. Dermal skeleton of the stem osteichthyan *Ligulalepis* from the Lower Devonian of New South Wales (Australia). Spanish Journal of Palaeontology, 38(1): 23–35.

Chang, M.M. and Smith, M.M., 1992. Is *Youngolepis* a porolepiform? Journal of Vertebrate Paleontology, 12(3): 294–312.

Cui, X., Qiao, T., Peng, L. and Zhu, M., 2025. New material of the Early Devonian sarcopterygian *Styloichthys changae* illuminates the origin of cosmine. Journal of Systematic Palaeontology, 23(1): 2432273.

Dearden, R.P., den Blaauwen, J.L., Sansom, I.J., Burrow, C.J., Davidson, R.G., Newman, M.J., Ko, A. and Brazeau, M.D., 2021. A revision of *Vernicomacanthus* Miles with comments on the characters of stem‐group chondrichthyans. Papers in Palaeontology, 7(4): 1949–1976.

Denison, 1979. Acanthodii. In: Schultze H-P, ed. Handbook of paleoichthyology. Vol. 5. Stuttgart: Gustav Fischer Verlag, 1–62. (Nostolepis).

Downs, J.P. and Donoghue, P.C., 2009. Skeletal histology of *Bothriolepis canadensis* (Placodermi, Antiarchi) and evolution of the skeleton at the origin of jawed vertebrates. Journal of Morphology, 270(11): 1364–1380.

Gagnier, P.Y., Hanke, G.F. and Wilson, M.V., 1999. *Tetanopsyrus lindoei* gen. et sp. nov., an Early Devonian acanthodian from the Northwest Territories, Canada. Acta Geologica Polonica, 49(2): 81–96.

Gardiner, B.G., 1984. The relationships of the palaeoniscid fishes, a review based on new specimens of *Mimia* and *Moythomasia* from the Upper Devonian of Western Australia. Bulletin of the British Museum (Natural History), Geology Series, 37(4): 173–428.

Giles, S., Rücklin, M. and Donoghue, P.C., 2013. Histology of “placoderm” dermal skeletons: Implications for the nature of the ancestral gnathostome. Journal of Morphology, 274(6): 627–644.

Gross, W., 1935. Histologische Studien am Aussenskelett fossiler Agnathen und Fische. Palaeontographica Abt. A, 83: 1–60.

Gross, W., 1956. Über Crossopterygier und Dipnoer aus dem baltischen Oberdevon im Zusammenhang einer vergleichenden Untersuchung des Porenkanalsystems paläozoischer Agnathen und Fische. Kungliga Svenska Vetenskapsakademiens Handlingar, (4)5(6): 1–140.

Gross, W., 1969. *Lophosteus* superbus Pander, ein Teleostome aus dem Silur Oesels. Lethaia, 2(1): 15–47.

Hanke, G.F., 2008. *Promesacanthus eppleri* n. gen., n. sp., a mesacanthid (Acanthodii, Acanthodiformes) from the Lower Devonian of northern Canada. Geodiversitas, 30(2): 287–302.

Hanke, G.F. and Davis, S.P., 2008. Redescription of the acanthodian *Gladiobranchus probaton* Bernacsek & Dineley, 1977, and comments on diplacanthid relationships. Geodiversitas, 30(2): 303–330.

Hanke, G.F. and Davis, S.P., 2012. A re-examination of *Lupopsyrus pygmaeus* Bernacsek & Dineley, 1977 (Pisces, Acanthodii). Geodiversitas, 34(3): 469–487.

Hanke, G.F. and Wilson, M.V., 2006. Anatomy of the Early Devonian acanthodian *Brochoadmones milesi* based on nearly complete body fossils, with comments on the evolution and development of paired fins. Journal of Vertebrate Paleontology, 26(3): 526–537.

Jerve, A., Qu, Q., Sanchez, S., Blom, H. and Ahlberg, P.E., 2016. Three-dimensional paleohistology of the scale and median fin spine of *Lophosteus superbus* (Pander 1856). PeerJ, 4: e2521.

Long, J., Barwick, R. and Campbell, K., 1997. Osteology and Functional Morphology of the Osteolepiform Fish.

Lu, J., Giles, S., Friedman, M., den Blaauwen, J.L. and Zhu, M., 2016. The oldest actinopterygian highlights the cryptic early history of the hyperdiverse ray-finned fishes. Current Biology, 26(12): 1602–1608.

MEEMANN, C. and Min, Z., 1993. A new middle Devonian osteolepidid from Qujing, Yunnan. Memoirs of the Association of Australasian Palaeontologists, 15: 183–198.

Mondéjar‐Fernández, J., Meunier, F.J., Cloutier, R., Clément, G. and Laurin, M., 2021. A microanatomical and histological study of the scales of the Devonian sarcopterygian *Miguashaia bureaui* and the evolution of the squamation in coelacanths. Journal of Anatomy, 239(2): 451–478.

Newman, M.J., Burrow, C.J., Den Blaauwen, J.L. and Davidson, R.G., 2014. The Early Devonian acanthodian *Euthacanthus macnicoli* Powrie, 1864 from the Midland Valley of Scotland. Geodiversitas, 36(3): 321–348.

Nian Zhong, W., Donoghue, P.C., Smith, M.M. and Sansom, I.J., 2005. Histology of the galeaspid dermoskeleton and endoskeleton, and the origin and early evolution of the vertebrate cranial endoskeleton. Journal of Vertebrate Paleontology, 25(4): 745–756.

O'Shea, J., Keating, J.N. and Donoghue, P.C., 2019. The dermal skeleton of the jawless vertebrate *Tremataspis mammillata* (Osteostraci, stem‐Gnathostomata). Journal of morphology, 280(7): 999–1025.

Qu, Q., Sanchez, S., Zhu, M., Blom, H. and Ahlberg, P.E., 2017. The origin of novel features by changes in developmental mechanisms: ontogeny and three‐dimensional microanatomy of polyodontode scales of two early osteichthyans. Biological Reviews, 92(2): 1189–1212.

Qu, Q., Zhu, M. and Wang, W., 2013. Scales and dermal skeletal histology of an early bony fish *Psarolepis romeri* and their bearing on the evolution of rhombic scales and hard tissues. PloS one, 8(4): e61485.

Schultze, H.P., 1968. Palaeoniscoidea-schuppen aus dem Unterdevon Australiens und Kanadas und aus dem Mitteldevon Spitzbergens. (No Title).

Schultze, H.P., 2016. Scales, enamel, cosmine, ganoine, and early osteichthyans. Comptes Rendus Palevol, 15(1-2): 83–102.

Schultze, H.P., Lawrence and Norman, J.Z., 1982. Ein primitiver acanthodier (Pisces) aus dem Unterdevon Lettlands. Paläontologische Zeitschrift, 56: 95–105.

Soler‐Gijón, R., 1999. Occipital spine of *Orthacanthus* (Xenacanthidae, Elasmobranchii): structure and growth. Journal of Morphology, 242(1): 1–45.

Turner, S., Burrow, C.J. and Warren, A., 2005. *Gyracanthides hawkinsi* sp. nov.(Acanthodii, Gyracanthidae) from the Lower Carboniferous of Queensland, Australia, with a review of gyracanthid taxa. Palaeontology, 48(5): 963–1006.

Valiukevicius, J., 1992. First articulated *Poracanthodes* from the Lower Devonian of Severnaya Zemlya. Fossil fishes as living animals, 1: 193–213.

Zhu, M., Yu, X. and Ahlberg, P.E., 2001. A primitive sarcopterygian fish with an eyestalk. Nature, 410(6824): 81–84.

Zhu, M., Yu, X., Wang, W., Zhao, W. and Jia, L., 2006. A primitive fish provides key characters bearing on deep osteichthyan phylogeny. Nature, 441(7089): 77–80.

Zhu, M., Zhao, W., Jia, L., Lu, J., Qiao, T. and Qu, Q., 2009. The oldest articulated osteichthyan reveals mosaic gnathostome characters. Nature, 458(7237): 469–474.

Zylberberg, L., Meunier, F.J. and Laurin, M., 2010. A microanatomical and histological study of the postcranial dermal skeleton in the Devonian sarcopterygian *Eusthenopteron foordi*. Acta Palaeontologica Polonica, 55(3): 459–470.

Zylberberg, L., Meunier, F.J. and Laurin, M., 2015. A microanatomical and histological study of the postcranial dermal skeleton of the Devonian actinopterygian *Cheirolepis canadensis*. Acta Palaeontologica Polonica, 61(2): 363–376.
