# Supplementary material for: The Dermal Skeleton of Stem‐Actinopterygian Moythomasia durgaringa and Its Implications for the Nature of the Ancestral Osteichthyan
Source: J Morphol. 2026 Mar 19;287(3):e70120. doi: 10.1002/jmor.70120 (PMC13003200; doi:10.1002/jmor.70120)
Supplement: Supplementary file 2 — S2. [file JMOR-287-e70120-s002.docx]

**Supplementary Dataset 2. Data and Scripts for Ancestral State Estimation**

This Supplementary Dataset contains phylogenetic trees, discrete character matrices, tip‐age datasets, and the R script used to perform the Structured Markov Model (SMM) ancestral state estimation for this study.

All files are openly available at the following repository:

<https://github.com/XianrenShan989/Supplementary-Dataset-for-ASE-Moythomasia>
